# Topological features of functional brain networks and subclinical impulsivity: an investigation in younger and older adults

**DOI:** 10.1007/s00429-023-02745-5

**Published:** 2024-03-06

**Authors:** Silvia Fornaro, Arianna Menardi, Antonino Vallesi

**Affiliations:** 1 Department of Neuroscience (DNS), University of Padova, Padova, Italy; 2 Padova Neuroscience Center, University of Padova, Padova, Italy

**Keywords:** Impulsivity, Graph-theory, Topology, Functional brain networks, Resting-state

## Abstract

Impulsive traits (i.e., the tendency to act without forethought regardless of negative outcomes) are frequently found in healthy populations. When exposed to risk factors, individuals may develop debilitating disorders of impulse control (addiction, substance abuse, gambling) characterized by behavioral and cognitive deficits, eventually leading to huge socioeconomic costs. With the far-reaching aim of preventing the onset of impulsive disorders, it is relevant to investigate the topological organization of functional brain networks associated with impulsivity in sub-clinical populations. Taking advantage of the open-source LEMON dataset, we investigated the topological features of resting-state functional brain networks associated with impulsivity in younger (*n* = 146, age: 20–35) and older (*n* = 61, age: 59–77) individuals, using a graph-theoretical approach. Specifically, we computed indices of segregation and integration at the level of specific circuits and nodes known to be involved in impulsivity (frontal, limbic, and striatal networks). In younger individuals, results revealed that impulsivity was associated with a more widespread, less clustered and less efficient functional organization, at all levels of analyses and in all selected networks. Conversely, impulsivity in older individuals was associated with reduced integration and increased segregation of striatal regions. Speculatively, such alterations of functional brain networks might underlie behavioral and cognitive abnormalities associated with impulsivity, a working hypothesis worth being tested in future research. Lastly, differences between younger and older individuals might reflect the implementation of age-specific adaptive strategies, possibly accounting for observed differences in behavioral manifestations. Potential interpretations, limitations and implications are discussed.

**Supplementary Information:**

The online version contains supplementary material available at 10.1007/s00429-023-02745-5.

## Introduction

Impulsivity is a multidimensional construct describing a tendency to act without forethought in response to internal or external stimuli, regardless of potential negative outcomes (Moeller et al. 2001; Dalley and Robbins 2017). Impulsive traits, when combined with environmental predisposing factors (e.g., Albertella et al. 2021), might lead to the implementation of risky behavioral strategies and, eventually, to the development of severe and debilitating addictive disorders characterized by a plethora of cognitive and behavioral abnormalities (Lee et al. 2019; Maxwell et al. 2020). If overlooked and untreated, impulsive traits and related disorders can deeply jeopardize the functioning of affected individuals and their significant others, with huge costs and consequences at the socio-economic and personal level (Birnbaum et al. 2011; Latvala et al. 2019; Manthey et al. 2021).

Interestingly, clinical populations characterized by impulsivity exhibit considerable differences in terms of typical age of onset and clinical presentations. These populations span from children and adolescents diagnosed with neurodevelopmental disorders (Ahmadi et al. 2021; Kumar et al. 2022; Zhang et al. 2020), to substance users (Motzkin et al. 2014; Wilcox et al. 2019), to older adults diagnosed with neurodegenerative disorders (Esteban-Penalba et al. 2021; Koh et al. 2020; Zhao et al. 2019). These groups have been found to share cognitive control impairments, frequently linked to specific functional brain abnormalities. Nonetheless, the heterogeneity of the aforementioned disorders—in terms of developmental trajectories, clinical manifestations, underlying pathogenetic mechanisms and pharmacological interventions—hinders the identification of transdiagnostic neural endophenotypes uniquely associated with impulsive traits and behaviors, either in the premorbid or in the chronic phases. Importantly, age might represent a confounding factor in the study of impulsivity, eventually jeopardizing the identification of its unique endophenotypes. Indeed, age can affect both functional connectivity and the topological organization of functional brain networks, cognitive control functions and behavior. Nonetheless, evidence concerning age-related differences in decision-making, impulsivity and risk-taking is mixed and often leading to inconsistent conclusions (e.g., Burnett et al. 2010; Kray et al. 2021; Leijenhorst et al. 2008; Paulsen et al. 2012). Moreover, age-dependent differences and changes in functional connectivity and in the topology of brain networks across lifespan have been extensively reported (Chong et al. 2019; Geerligs et al. 2015; Meunier et al. 2009; Puxeddu et al. 2020; Song et al. 2014). Therefore, age might represent a massive source of systematic (and co-variating) noise when trying to disentangle impulsivity-related from age-related brain changes and endophenotypes.

Concerning age-related differences in topological organization of brain networks associated with impulsivity, previous studies mainly focused on abnormalities in whole-brain functional connectivity and/or in canonical brain networks, predominantly in pathological populations (e.g., Chen et al. 2021; Hege et al. 2015; Tessitore et al. 2017; Whelan et al. 2012). Notably, very few studies tried to characterize topological features and organizational properties of functional brain networks related to impulsivity (e.g., Davis et al. 2013; Gell et al. 2023) and age was rarely included as a potential confounding factor.

Recently, significant advancements in recognizing distinct neural networks responsible for several behavioural and cognitive manifestations related to impulsivity—spanning from impaired response inhibition and risky decision-making to the intolerance of delayed rewards—have been made. Specifically, reciprocal interactions between frontal circuits, striatal and limbic regions were proposed to account for clinical manifestations and executive dysfunction related to impulsivity (Coccaro et al. 2011; Dalley et al. 2011; Dalley and Robbins 2017; Xu et al. 2021).

Nevertheless, to date, trait impulsivity in healthy individuals (Reynolds et al. 2019) and its relationship with topological abnormalities of functional networks, especially from a graph-theoretical perspective (Davis et al. 2013), has been poorly investigated. Indeed, singling out specific transdiagnostic functional endophenotypes of impulsivity from a topological viewpoint is fundamental for an early identification of at-risk populations and for developing effective prevention strategies. Henceforth, investigating the association between trait impulsivity and topological features of functional networks classically associated with impulsivity traits in healthy individuals might shed light on the neural mechanisms subtending impulsive disorders and related cognitive deficits, usually identified and diagnosed when chronicization has already occurred.

In the present study, we hypothesized that trait impulsivity might be associated with topological alterations of functional networks, visible even at rest, that is, when the individual is not engaging in any active task execution. Given the limited empirical evidence, we adopted an exploratory approach aimed at thoroughly describing the relationships between behavioral measures of impulsivity (i.e., UPPS Impulsive Behavior scale, Whiteside and Lynam 2001) and graph-theoretical indices of segregation, integration and efficiency. Graph measures were derived in consideration of an a priori selected network that is classically associated with impulsivity (Coccaro et al. 2011; Dalley et al. 2011; Dalley and Robbins 2017; Xu et al. 2021), along with its constituting sub-networks (i.e., frontal, limbic and striatal modules), and its subcomponents taken individually (i.e., 14 frontal, 16 limbic, and 8 striatal nodes). We also hypothesized that—due to adaptation mechanisms and/or the chronicization of impulsive traits—age might play a role in the reconfiguration of the topology of functional networks associated with impulsivity. We analyzed two samples of healthy younger and older individuals, whose resting state functional magnetic resonance imaging (fMRI) data are made openly available from the LEMON dataset (http://fcon_1000.projects.nitrc.org/indi/retro/MPI_LEMON.html, Babayan et al. 2019). Therefore, we expected resting-state functional topological features to differ between healthy younger and older populations.

## Materials and methods

### Participants

Participants were selected from the “Max Planck Institute Leipzig Mind-Brain-Body Dataset LEMON” (Babayan et al. 2019), an open-source dataset including subjects recruited between 2013 and 2015 at the University of Leipzig (Germany). Data were collected in accordance with the Declaration of Helsinki and the protocol of the original study was approved by the ethical committee of the medical faculty of the University of Leipzig.

The final sample included 207 subjects, divided into two distinct age groups of n = 146 (age range 20–35) and n = 61 (age range 59–77), respectively. Demographic data of both samples are reported in Table 1.

**Table 1:** Demographic data of younger and older individuals

	Age	Sex	Years of education	Handedness	Smoking habits
	*M (SD)*	*F (M)*	*M (SD)*	*R (L, AMB)*	*S (NS)*
*Younger*	25.51 (3.40)	45 (101)	12.66 (1.35)	127 (17, 2)	29 (117)
*Older*	67.75 (5.12)	34 (27)	10.23 (2.42)	57 (2, 2)	5 (56)

*R* right-handed, *L* left-handed, *AMB* ambidextrous, *S* smokers, *NS* non-smokers

### Behavioral measures

The UPPS Impulsive Behaviour scale (Whiteside and Lynam 2001) was administered to measure impulsive tendencies and behaviors. A validated version of the 45-item scale, based on a four-factor model of impulsivity (Whiteside and Lynam 2001; Kämpfe and Mitte 2009), was administered in German (Schmidt et al. 2008). The scale includes four subscales: (1) *urgency*: tendency to experience strong impulses, often accompanied by negative affect (e.g., “In the heat of an argument, I will often say things that I later regret.”; α = 0.82); (2) *lack of premeditation*: difficulty to understand and think about the consequences of an act before doing so (e.g., “I usually make up my mind through careful reasoning.”; α = 0.80); (3) *lack of perseverance*: inability to focus on difficult tasks (e.g., “Once I start a project, I almost always finish it.”; α = 0.85); and (4) *sensation-seeking*: tendency to engage in exciting activities and being open to trying new experiences that can be dangerous (e.g., “I welcome new and exciting experiences and sensations, even if they are a little frightening and unconventional.”; α = 0.83). Scores on a 4-point Likert scale ranged from 1 (strongly agree) to 4 (strongly disagree).

Descriptive statistics of UPPS subscales for both younger and older participants are reported in Table 2. Higher values of UPPS scores represent greater impulsivity traits/behaviors.

**Table 2:** Descriptive statistics of UPPS scores for younger and older individuals

	Urgency	Lack of premeditation	Lack of perseverance	Sensation-seeking
	*M (SD)*	*M (SD)*	*M (SD)*	*M (SD)*
*Younger*	26.39 (4.98)*	22.82 (4.08)*	20.06 (4.92)**	34.65 (6.44)**
*Older*	24.52 (4.32)	21.55 (3.67)	17.15 (3.47)	27.21 (6.09)

T-tests were performed to compare UPPS scores between the two samples

*p < 0.05; **p < 0.0001

### MRI data acquisition

Structural and functional MRI data were acquired with a 3 Tesla MRI scanner (Verio, Siemens Healthcare GmbH). During the acquisition, subjects were asked to remain awake with their eyes open and to fixate on a low-contrast fixation cross. For our analyses, we considered BOLD resting state fMRI scans, using T2-weighted multiband EPI* sequence (TR = 1400 ms, TE = 30 ms, flip angle = 69°, echo spacing = 0.67 ms, number of volumes = 657, voxel size (isotropic) = 2.3 mm, slices per volume = 64, total acquisition time = 15 min 30 s) and T1-weighted structural volumes acquired using MP2RAGE sequence (TR = 5000 ms, TE = 2.92 ms, TI1 = 700 ms, TI2 = 2500 ms, FOV = 256 mm, isotropic voxel size = 1 mm^3^). The structural volumes were acquired with 176 slices interspersed during 8 min and 22 s of scanning.

### Neuroimaging analyses

#### Preprocessing

All processing steps were performed in FSL (Jenkinson et al. 2012). The following pre-statistics processing was applied: motion correction using MCFLIRT (Jenkinson et al. 2002); non-brain removal using BET (Smith 2002); spatial smoothing using a Gaussian kernel of FWHM 6.0 mm; grand-mean intensity normalization of the entire 4D dataset by a single multiplicative factor; high-pass temporal filtering (Gaussian-weighted least-squares straight line fitting, with sigma = 50.0 s). Distortion correction was performed using TOPUP (Smith et al. 2004). FLIRT was used to coregister each participant’s functional and anatomical volume using the normalized mutual information as a cost function and 6-degree-of-freedom. Finally, the brain was parcelled into 200 cortical regions of interest (ROIs) according to the Schaefer Atlas (Schaefer et al. 2018) and an additional 16 ROIs were included according to the Melbourne Subcortex Atlas (Tian et al. 2020), making a total of 216 ROIs. In particular, we decided to employ the Schaefer atlas as it was conceived based on a gradient-weighted Markov Random Field (gwMRF) model, integrating both local gradient and global similarity approaches, thus generating parcels that are both neurobiological meaningful as well as useful for applications requiring dimensionality reduction (Schaefer et al. 2018). As a result, Schaefer’s parcellation has been proven to be more homogeneous than other parcellations, and it agrees with boundaries of certain cortical areas defined using histology and visuotopic fMRI (Schaefer et al. 2018). Another advantage of the Schaefer atlas is that it also divides parcels according to the canonical 7-networks classification (Yeo et al. 2011). To test the consistency of our findings, analyses were re-run using a different parcellation scheme, according to the Yeo 7-networks atlas (Yeo et al. 2011), consisting of 51 ROIs. Results obtained with this alternative parcellation scheme are reported in the Supplementary Materials.

Finally, functional connectivity matrices were computed from Pearson's correlation between all pairs of ROIs’ functional time-series. A Fisher’s z transformation was then applied to normalize the data and ease the interpretation of correlation strengths.

### Graph-theory measures

Several graph-theoretical measures were computed within the a priori selected regions associated with impulsivity (38 nodes) considered altogether, as well as separately (14 frontal nodes, 16 limbic nodes, 8 striatal nodes; see Supplementary materials Table S1 for a complete list). Finally, we averaged together nodal measures belonging to each cluster (frontal, limbic and striatal) to characterize each sub-network in terms of its specific topological functional organization. To decrease the risk of false positives in identifying significant connections, graph theory measures were extracted from the individual adjacency matrices obtained by applying a stringent 80% threshold (i.e., only the strongest 20% of connections within the connectivity matrix were retained. For a description of the robustness across different thresholding procedures, see Supplementary Materials). Measures were computed using the Brain Connectivity Toolbox (Whitfield-Gabrieli and Nieto-Castanon 2012) implemented in MATLAB (2023a). To better characterize the mechanisms of information flow in the brain, we extracted both indices of integration and segregation, as follows.

Integration indices:

1. *characteristic path length*: average minimum number of steps needed to reach all pairs of nodes in the network;
2. *global efficiency*: average inverse shortest path length in the network, which quantifies the easiness of information sharing at the global network level;
3. *eccentricity*: maximal shortest path length between any pair of nodes in a network;
4. *diameter*: maximum distance in the network.
5. *radius*: minimum distance in the network.

Higher values at these metrics indicate greater integration of the information at the network level. The only exception is represented by the measure of characteristic path length, for which lower indices are indicative of shorter communication distance between nodes, hence higher integration.

Segregation indices:

(6) *modularity*: statistics quantifying the degree to which the network may be subdivided into clearly defined modules, based on a greater distribution of within-module, rather than between-modules, connections;
(7) *clustering coefficient*: fraction of nodes’ neighbours that are neighbours to each other, reflecting how densely connected is the network;
(8) *local efficiency*: equal to the global efficiency computed on node neighbourhoods; For all these measures, higher values are indicative of greater segregation within the system.

Furthermore, a *small-worldness* index was ultimately extracted as a general measure of efficiency in the graph, describing the extent to which the network is characterized by concomitant high clustering and low path length.

Finally, specific graph-theoretical measures were extracted at the node-level for all the selected ROIs, specifically: (9) *degree* (i.e., number of a node’s connections)*;* (10) *clustering coefficient*; (11) *eccentricity;* (12) *local efficiency;* and (13) *participation coefficient* (i.e., measure of diversity of intermodular connections of individual nodes).

### Statistical analyses

Statistical analyses were performed with MATLAB software (R2023a), for both samples of younger and older participants. Analyses were performed hierarchically, starting at the network-level (i.e., 38-nodes impulsivity network), then considering its sub-networks separately (i.e., frontal, limbic and striatal modules), and finally considering nodal metrics computed separately for each selected ROI. Non-parametric Spearman correlations between graph theoretical measures computed for the network (and its components) classically associated with impulsivity (Coccaro et al. 2011; Dalley et al. 2011; Dalley and Robbins 2017; Xu et al. 2021) and UPPS scores were performed, as the assumption of normality was not always fulfilled. Correction for multiple comparisons was applied to decrease the risk of false positives using the False Discovery Rate (FDR). To reach a deeper level of understanding, the relationship between UPPS scores and the topological properties of single nodes forming the selected network (38 ROIs) was assessed. Bonferroni-Holmes correction was applied considering the increased risk of false positives, given the higher number of statistical tests performed. Furthermore, linear mixed-effects models were performed to investigate if impulsivity scores could be predicted by nodal measures considered together and averaged between nodes belonging to the same network. To account for potential confounding effects, age, sex, education and smoking habits were included in the models as random factors. Nonetheless, none of the latter showed a significant effect, therefore these were not included in the final models. Outliers in the models were identified as those with more than three scaled median absolute deviations (MAD) from the median (n = 11 and n = 9 individuals for the younger and the older cohorts, respectively) and removed from the analyses. Finally, to test for significant differences in network-level brain-behavior relationships between younger and older individuals, we performed a series of Fisher's Z tests to compare correlations between independent samples (Diedenhofen and Musch 2015; for more details see the dedicated paragraph in the “Results” section).

## Results

### Younger adults

#### Network-level analyses

**Fig. 1:**
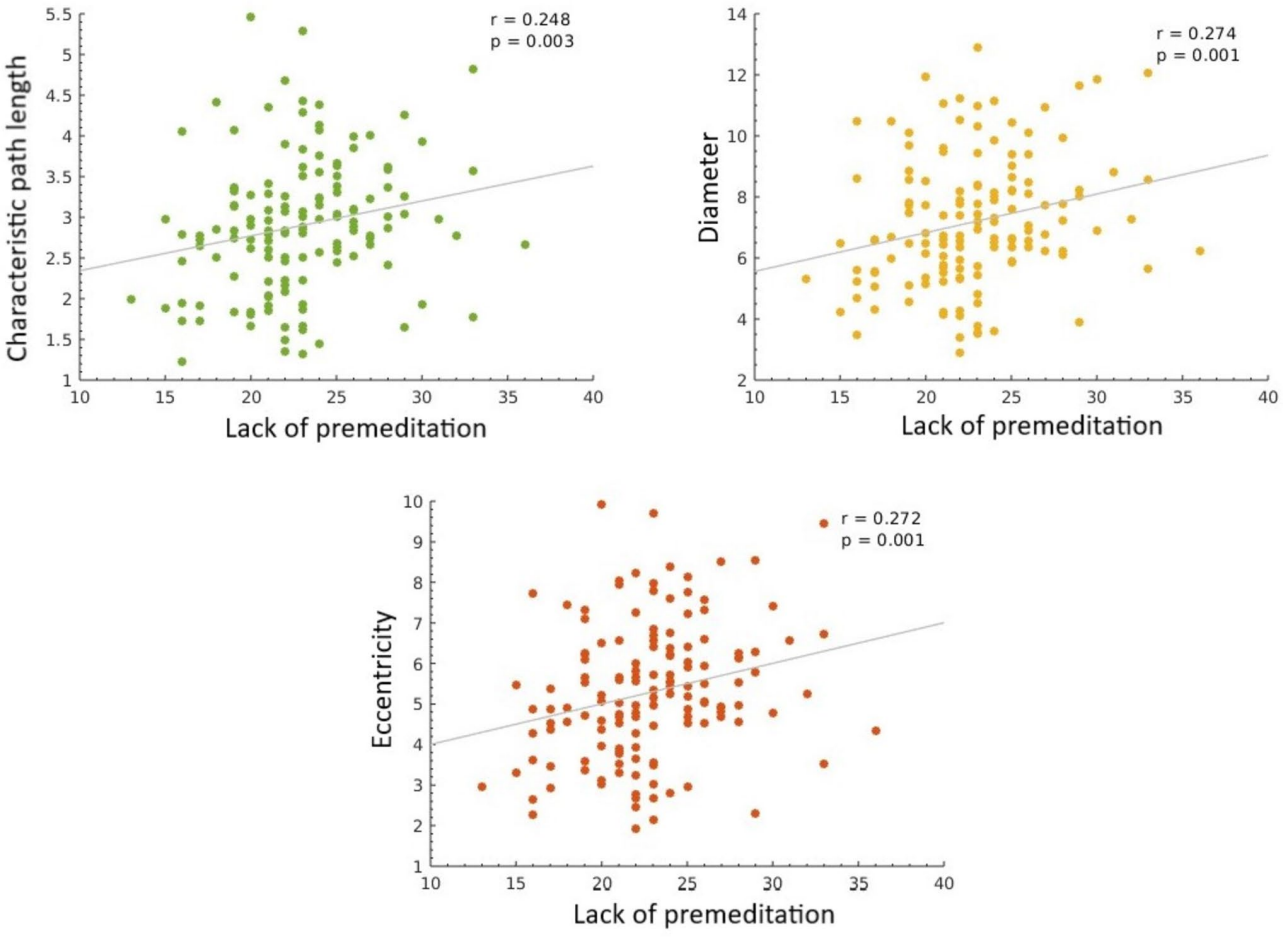
Significant associations (FDR-corrected) between graph-theoretical measures computed at the network level (all selected nodes considered altogether) and UPPS scores for younger individuals

Spearman correlations revealed that lack of premeditation scores positively correlated with integration indices such as characteristic path length (r = 0.248, *p* = 0.0029), diameter (r = 0.274, *p* < 0.001) and eccentricity (r = 0.272, *p* = 0.001) at the network-level (Fig. 1).

**Fig. 2:**
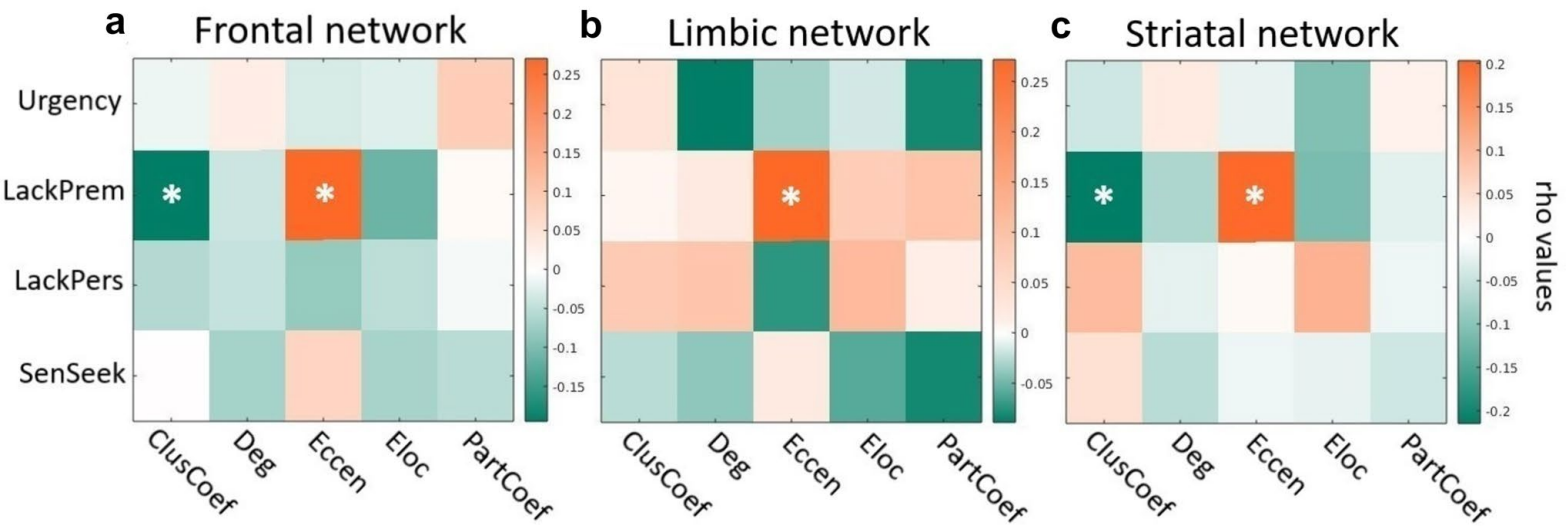
Significant associations between graph-theoretical measures computed at the network level (nodal measures mediated for nodes belonging to the three selected networks, separately) and UPPS subscales for younger individuals. **A** Correlations for mean frontal network measures. **B** Correlations for mean limbic network measures. **C** Correlations for mean striatal network measures. *ClusCoef* clustering coefficient, *Deg* degree, *Ecc* eccentricity, *Eloc* local efficiency, *LackPrem* lack of premeditation, *LackPers* lack of perseverance, *PartCoef* participation coefficient, *SenSeek* sensation-seeking

Moreover, we observed a significant positive correlation between lack of premeditation scores and eccentricity of nodes belonging to the frontal (r = 0.270, *p* = 0.0011), limbic (r = 0.272, *p* = 0.0011) and striatal (r = 0.203, *p* = 0.0153) networks, while striatal clustering coefficient negatively correlated with lack of premeditation scores (r = – 0.215, p = 0.0104). All results were corrected for multiple comparisons (FDR-corrected *p* < 0.05; Fig. 2).

#### Node-level analyses

**Fig. 3:**
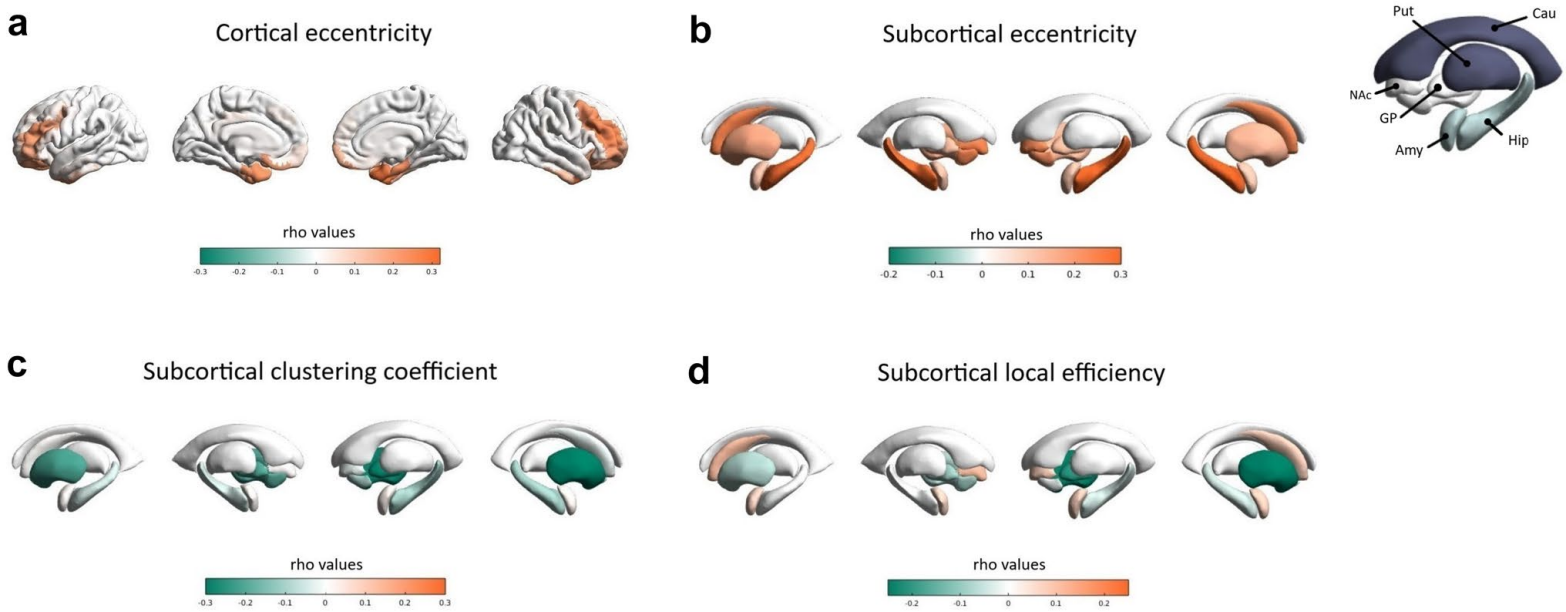
Significant associations between nodal measures of selected nodes and *lack of premeditation* scale in younger individuals (Bonferroni-corrected). **A** Associations between lack of premeditation scores and eccentricity of cortical nodes. **B** Associations between lack of premeditation scores and eccentricity of subcortical nodes. **C** Associations between lack of premeditation scores and clustering coefficient of subcortical nodes. **D** Associations between lack of premeditation scores and local efficiency of subcortical nodes. In the upper right corner, a depiction of selected subcortical ROIs and related labels. *Amy* amygdala, *Cau* caudate, *GP* globus pallidus, *Hip* hippocampus, *NAc* nucleus accumbens, Put putamen

A consistent positive association between eccentricity of frontal (mainly right-sided), limbic (bilateral) and striatal (right-sided) nodes and lack of premeditation emerged. Moreover, lack of premeditation was negatively associated with both nodal clustering coefficient and local efficiency of right-sided striatal nodes (Fig. 3). Considering the higher number of correlations performed for the nodal analyses, a more stringent correction for multiple comparisons was applied (Bonferroni-Holmes). Coordinates of nodes surviving correction for multiple comparisons and related statistics are reported in Table 3.

**Table 3:** Significant Spearman correlations surviving Bonferroni-correction for multiple comparisons (p < 0.0013) between nodal measures and the UPPS subscale *“lack of premeditation”*

	MNI			Region	rho	*p* (corrected)
	*x*	*y*	*z*			
*Clustering coefficient*	20.12	– 3.82	– 1.70	GP_right	– 0.30461539	0.008
	26.79	0.44	0.83	PUT_right	– 0.32159511	0.004
*Local efficiency*	26.79	0.44	0.83	PUT_right	– 0.26910249	0.046
*Eccentricity*	– 32	42	– 14	OFC_orb_left	0.27280513	0.032
	30	58	4	antPFC_right	0.29847158	0.011
	30	48	28	dlPFC_right	0.27273717	0.032
	40	34	38	dlPFC_right	0.28743414	0.018
	42	14	48	FEF_right	0.26570837	0.04
	– 28	10	– 34	Tpole_left	0.29025068	0.016
	30	8	– 38	Tpole_right	0.30203075	0.01
	– 25.19	– 22.18	– 14.14	Hip_left	0.28405224	0.02
	27.19	– 22.18	– 14.14	Hip_right	0.2957648	0.013
	26	– 10	– 32	ParaHip_right	0.27870387	0.025

*antPFC* anterior prefrontal cortex, *dlPFC* dorsolateral prefrontal cortex, *FEF* frontal eye field, *GP* globus pallidus, *Hip* Hippocampus, *OFC_orb* orbitofrontal cortex pars orbitalis, *ParaHip* parahippocampal gyrus, *PUT* putamen, *Tpole* temporal pole

#### Linear mixed-effect models

LMMs were performed to test if UPPS subscales scores could be predicted from mean nodal network measures. Separate models were run for each UPPS subscale (urgency, lack of premeditation, lack of perseverance, sensation seeking) and network (frontal, limbic, striatal). Results revealed that lack of premeditation scores were significantly predicted by frontal (t = 2.2062, *p* < 0.05), limbic (t = 2.7062, *p* < 0.01) and striatal (t = 2.5141, *p* < 0.05) eccentricity, and by frontal participation coefficient (t = 2.0316, *p* < 0.05). Finally, sensation-seeking scores were significantly predicted by limbic eccentricity (t = 2.1531, *p* < 0.05).

### Older adults

#### Network and node level analyses

Notably, none of the Spearman brain-behavior correlations performed for older individuals survived to correction for multiple comparisons, at any level of analysis. For this reason, the latter results are not presented.

#### Linear mixed-effects models

LMMs revealed that lack of perseverance scores were significantly predicted by mean frontal clustering coefficient (t = – 2.117, *p* < 0.05), degree (t = – 2.687, *p* < 0.01), eccentricity (t = – 2.373, *p* < 0.05) and local efficiency (t = 2.903, *p* < 0.01), as well as by mean limbic participation coefficient (t = 2.249, *p* < 0.05), mean striatal eccentricity (t = – 2.239, *p* < 0.05) and local efficiency (t = 2.630, *p* < 0.05). Finally, lack of premeditation scores were significantly predicted by mean striatal eccentricity (t = – 2.089, *p* < 0.005).

### Comparison of brain-behavior associations between younger and older adults

**Fig. 4:**
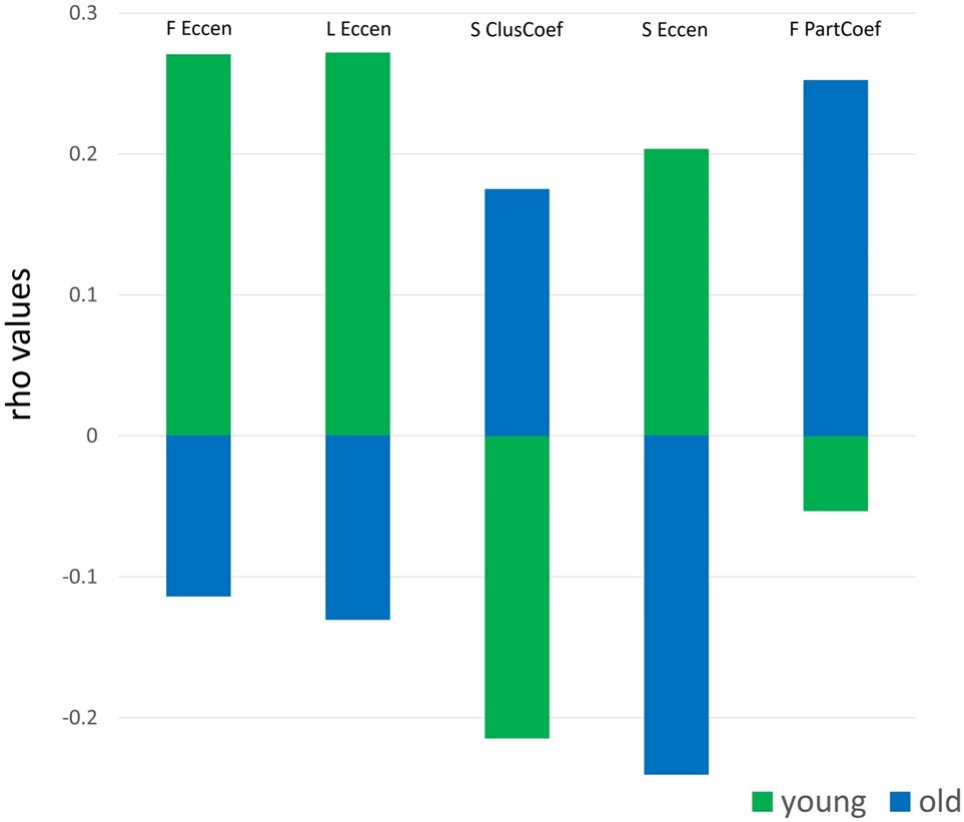
Significant differences in strength and direction of brain-behavior associations for mean network measures between younger and older individuals. *ClusCoef* clustering coefficient, *Eccen* eccentricity, *PartCoef* participation coefficient, *F* frontal, *L* limbic, *S* striatal

Finally, to directly test the differences of investigated brain-behavior associations between younger and older individuals, we performed a series of Fisher's Z tests to compare correlations between independent samples (Diedenhofen and Musch 2015). Specifically, we compared associations tested at the network-level. Significant differences in the strength and directionality of brain-behavior relationships between younger and older individuals were observed. Significant results were found for the associations between lack of premeditation and frontal (z = 2.503, *p* < 0.05), limbic (z = 2.619, *p* < 0.01), striatal (z = 2.882, *p* < 0.005) eccentricity, and striatal clustering coefficient (z = – 2.523, *p* < 0.05). Lastly, a significant difference emerged for the association between sensation-seeking and frontal participation coefficient (z = – 1.987, *p* < 0.05, see Fig. 4). Overall, associations characterized by significant differences showed an opposite pattern for younger versus older individuals. (Fig. 4 about here). Additionally, a sensitivity power analysis was performed in G*Power for testing the effect sizes of the Fisher's z tests performed to compare brain-behavior correlations between younger and older adults. Specifically, we tested the correlations between two independent Spearman's rho (i.e., younger adults, n = 146; older adults, n = 61) and we set the power at 0.80. Our analyses revealed a critical z of 1.96 and an effect size q of 0.44.

## Discussion

The aim of the present study was to investigate the relationship between impulsive traits and the topological organization of functional networks in healthy individuals using an exploratory approach, at the level of specific networks and nodes known to be involved in impulsive behaviors and found to be altered in clinical populations characterized by impulsivity. We also hypothesized that age might play a role in the modulation of the aforementioned relationships. Therefore, we tested these relationships separately for younger and older individuals. Results showed distinct patterns of such relationships for younger and older individuals, respectively, either at the network level or at the node level.

Specifically, impulsivity in younger individuals was found to be associated with a more widespread, less segregated and less efficient functional organization, either at the level of specific networks or at the level of specific nodes. Interestingly, *lack of premeditation* was the most characterizing dimension, as it was consistently found to be positively associated with integration measures and negatively associated with segregation/efficiency measures at all levels of analysis.

Moreover, for younger individuals at the single-node level, a specific integration index (i.e., eccentricity) characterizing several prefrontal nodes—mainly located in the right hemisphere—and bilateral temporal poles were found to be positively associated with lack of premeditation. On the other hand, segregation (i.e., clustering coefficient) and efficiency measures of striatal (right-sided) nodes negatively correlated with lack of premeditation. Such associations might speculatively reflect a major involvement of several right prefrontal subdivisions (for a complete list of prefrontal nodes involved, see Table 3) in widespread information processing and long-range communication between distant modules. Interestingly, lateral prefrontal cortices are thought to subserve different high-level cognitive functions (Goel 2019; Ravaja et al. 2013; Vallesi 2021). Indeed, the right prefrontal cortex is thought to play a key role in planning when dealing with dynamic events, as it integrates information in temporally-ordered sequences (Grafman et al. 2005; Kaller et al. 2011). Therefore, an increased involvement of right prefrontal nodes in widespread and long-range information processing might, in turn, hinder local information processing at the level of prefrontal modules and local circuits. This might lead to defective sequencing of information and, eventually, to the impossibility of precisely representing the dynamics regulating temporally-ordered events and how such events would evolve and could be influenced by one’s own actions.

Secondly, concerning findings about limbic nodes, structural alterations of the bilateral temporal pole was found to be positively associated with trait impulsivity (Fineberg et al. 2014; Liu and Feng 2017; Schilling et al. 2013; Pan et al. 2021). The temporal pole plays a crucial role in processing sensory inputs and emotional stimuli, alongside other limbic regions. Notably, the ability to manage negative emotions can deeply influence how action goals and outcomes are represented (Olson et al. 2007; Van Overwalle and Baetens 2009). Evidence about the functions of the temporal pole suggests that it plays a fundamental role in restraining social behaviors and in dealing with negative emotions contributing to impulsive decision-making (Bornovalova, et al. 2005; Garon and Moore 2006). Therefore, increased eccentricity of the temporal poles within the cortico-striatal-limbic circuit might, speculatively, reflect an increased involvement of emotional processing in implementing decision-making. Alternatively, it might underlie a compensatory mechanism aimed at dealing with and regulating overwhelming negative emotions, possibly reflecting—at a behavioral level—an attempt to limit impulsive responses and actions.

Lastly, concerning findings about striatal nodes (i.e., decreased efficiency and clustering associated with lack of premeditation), integrity of striatal and fronto-striatal circuits is thought to play a crucial role in cognitive flexibility and goal-directed behavior, either in preclinical or in clinical conditions (Middleton and Strick 2000; Vaghi et al. 2017). Interestingly, there is evidence that the striatum (i.e., caudate and dorsomedial striatum) plays a role in integrating reward-related information needed to implement action control (Balleine et al 2007a) and, thus, decision-making (Balleine et al 2007b), along with prefrontal circuits. According to the Dual Systems Theory, action control is implemented by balancing habitual and goal-directed systems (Doñamayor et al. 2022), both subserved by cortico-striatal networks. Evidence suggests that populations characterized by impulsivity, such as substance users, show a shift towards habitual relative to goal-directed strategies when implementing specific behaviors (Ersche et al. 2021). Therefore, a decreased functional segregation and efficiency of striatal nodes might reflect a defective balance in information processing within striatal modules, with a deficit in implementing goal-directed strategies and an increased reliance on long-established stimulus–response associations (i.e., habits).

To sum up, a more widespread and less efficient functional organization of brain networks at rest in younger individuals is characterized by difficulties in foreseeing the consequences of one’s own actions (i.e., lack of premeditation) and might reflect a shift towards global information processing, possibly associated with an impairment in local information processing. In other words, information might be projected to distant modules before being efficiently processed at the local level, possibly leading to a loss of functional specialization and precision (i.e., increased uncertainty of predictions and associated representations; Soltani and Koechlin 2022). This might lead to an inability to correctly predict the consequences of one’s own actions (i.e., impaired learning of action-outcome associations) and, eventually, might favour the implementation of habitual over goal-directed behavioral strategies (Lee et al. 2014; Soltani and Koechlin 2022). The observed association between more widespread and less clusterized organization and the lack of premeditation interested nodes belonging to frontal, limbic and striatal networks. These circuits are known to be involved in the implementation of impulsive behaviors (Coccaro et al. 2011; Hobkirk et al. 2019; Quaglieri et al. 2020; Xu et al. 2021). As a result, they have also been consistently found to be functionally and structurally altered in clinical populations characterized by impulsivity (Inuggi et al. 2014; Koh et al. 2020; Oliva et al. 2020; Quaglieri et al. 2020; Reynolds et al. 2019; Wang et al. 2016), and are marked by specific neurotransmitter profiles possibly underlying impulsive behaviors (Dalley and Robbins 2017; Hammes et al. 2019; Gell et al. 2023). Therefore, we speculate that our findings about reduced efficiency paralleled by a more global pattern of information processing in such networks might reflect the implementation of impulsive behaviors as a result of an inability to form accurate and precise representations of the consequences of one’s own actions. Indeed, healthy functional brain networks were found to be characterized by a rather modular and reduced widespread organization (Meunier et al. 2009; Ferrarini et al. 2009). Nevertheless, some evidence showed that impulsivity was associated with increased intra-modular connections and a decreased inter-modular connections at rest (Devis et al. 2013), suggesting that impulsivity might be subtended by a predominantly segregated organization of brain networks. Hence, further studies are needed to better characterize the topological features of functional brain networks underlying impulsivity.

Concerning the topological organization of functional networks in impulsive older individuals, correlational analyses did not survive correction for multiple comparisons, possibly owing to the relatively smaller sample size (n = 61) which might have decreased the statistical power and/or to the increased inter-individual variability of BOLD signal for older compared to younger individuals (D'Esposito et al. 2003; Grady and Garrett 2014). Nonetheless, LMMs revealed that impulsivity scores were significantly predicted by (1) increased segregation and local efficiency of the frontal and striatal networks, and by (2) increased participation of the limbic nodes. Speculatively, this might reflect a deficit in correctly processing and integrating emotional stimuli within circuits subserving emotion regulation. Notably, these circuits are known to be responsible for reward, emotional processing and regulation (Harada et al. 2021; Kebets et al. 2021; Molina-Ruiz et al. 2020, Morein-Zamir and Robbins 2015). Indeed, the effective processing and regulation of emotional stimuli is fundamental to properly implement goal-directed behaviors and to prevent negative consequences (Miller and Racine 2022; Pruessner et al. 2020). Therefore, older impulsive individuals might be characterized by deficits in emotional processing and regulation, eventually leading to deficits in implementing goal-directed behavioral strategies aimed at preventing negative outcomes. Nonetheless, the latter interpretations are speculative in nature, given the lack of robustness for correlational evidence and given the reduced sample size for the group including older individuals.

Interestingly, when the directions of relationships between impulsivity and graph-theory measures were explicitly compared between younger and older individuals, consistent opposite patterns emerged at the network level for all the tested associations, indeed supporting the hypothesis about the role of age in modulating the associations between topological functional abnormalities and impulsivity.

In other words, specular patterns for younger and older individuals emerged: positive associations between graph-theory indices and impulsivity in younger individuals were paralleled by negative associations in older individuals (frontal, limbic and striatal eccentricity), and vice versa (striatal clustering coefficient). This finding might indicate a key role of age in the topological reconfiguration of functional brain networks of impulsive individuals. In other words, mechanisms of functional adaptation might occur across the lifespan to account for topological functional alterations underlying impulsivity, thus allowing the development of alternative behavioral strategies to cope with daily challenges. Alternatively, such a pattern might reflect a progressive deterioration of functional brain networks associated with age. Nonetheless, given the age gap in the full sample, we were not able to perform more sophisticated statistical analyses with age as a continuous predictor. Moreover, our study was cross-sectional, which entails all the known limitations for this kind of experimental design (e.g., Levin 2006). Therefore, other studies need to be carried out (see limitations paragraph below) to deeply investigate the relationship between the topological functional reorganization of brain networks and impulsivity across the lifespan, if possible longitudinally, and to corroborate the role of age in mediating or moderating such relationships and in explaining their changes across the lifespan.

Our study—aimed at exploring the functional organization of brain networks associated with trait impulsivity—indeed entails some limitations that must be addressed in future studies. Firstly, our sample included only healthy individuals, and impulsivity values considered pathological were excluded from the analyses. Therefore, the generalizability of our findings from sub-clinical tracts to pathological populations characterized by impulsivity awaits further experimental confirmation. Secondly, the age gap in the full dataset between younger and older individuals prevented us from including age as a continuous covariate in our statistical models and, therefore, from carefully accounting for its effects, as it would be advisable given the above-discussed inconsistencies and gaps in the literature regarding age-related changes associated with impulsivity. Thirdly, the two tested populations largely differ in terms of sample size. Moreover, variance in BOLD signal is known to be intrinsically higher in older compared to younger adults (D'Esposito et al. 2003; Grady and Garrett 2014), which might have affected the results in unpredictable ways. Moreover, the arbitrariness of some methodological choices (e.g., statistical thresholding of adjacency matrices, parcellation procedure, a priori selection of nodes and networks) might have also affected the final results. Furthermore, from the methodological viewpoint, we ran our analyses considering 20% of the strongest connections. Nonetheless, the arbitrariness of such an approach entails that different results may emerge when different thresholds are considered (for results obtained with other thresholding methods, see Supplementary Materials). Indeed, our methodological choice may have affected our results in unpredictable ways. For instance, it might have left out relevant information (van Wijk et al. 2010), and/or it might have affected the computation of specific graph-theoretical metrics or group comparisons (van den Heuvel et al. 2017). An additional issue that needs to be considered is the risk of alterations in the functional connectome induced by residual motion artefacts despite the fact that data were carefully checked, which is an intrinsic limit of fMRI (Ciric et al. 2017; Lydon-Staley et al. 2019; Parkes et al. 2018). Lastly, the correlational and cross-sectional nature of our study prevents us from proposing any causal hypotheses or conclusions regarding either the relationships between impulsivity and the topological functional organization of brain networks or the role of age in mediating such relationships, above and beyond generation of new working hypotheses for further investigation. Future studies are indeed needed to clarify the directionality of the tested associations, as well as to longitudinally investigate and interpret changes of such associations across lifespan.

In conclusion, alterations in the topology of functional brain networks at rest might underlie specific behavioral and cognitive alterations associated with impulsive traits at the premorbid level. Moreover, differences in the topological features of functional brain networks associated with impulsivity between younger and older individuals might reflect adaptation mechanisms, possibly occurring across lifespan to cope with deficits in impulse control. Lastly, typical age-related changes in the topology of functional brain networks might account for differences in behavioral manifestations of impulsivity between younger and older individuals.

### Supplementary Information

Below is the link to the electronic supplementary material.

Supplementary file1 (DOCX 524 KB)

## Data Availability

The complete LEMON data, including raw and preprocessed functional and structural data, as well as demographic and behavioural data, can be accessed via the following link: http://fcon_1000.projects.nitrc.org/indi/retro/MPI_LEMON.html.

## References

[CR1] Ahmadi M, Kazemi K, Kuc K, Cybulska-Klosowicz A, Helfroush MS, Aarabi A (2021). Resting state dynamic functional connectivity in children with attention deficit/hyperactivity disorder. J Neural Eng.

[CR2] Albertella L, Rotaru K, Christensen E, Lowe A, Brierley ME, Richardson K, Yücel M (2021). The influence of trait compulsivity and impulsivity on addictive and compulsive behaviors during COVID-19. Front Psych.

[CR3] Babayan A (2019). A mind-brain-body dataset of MRI, EEG, cognition, emotion, and peripheral physiology in young and old adults. Sci Data.

[CR4] Balleine BW, Delgado MR, Hikosaka O (2007). The role of the dorsal striatum in reward and decision-making. J Neurosci.

[CR5] Balleine BW, Doya K, O'Doherty J, Sakagami M (2007). Reward and decision making in corticobasal ganglia networks.

[CR6] Birnbaum HG, White AG, Schiller M, Waldman T, Cleveland JM, Roland CL (2011). Societal costs of prescription opioid abuse, dependence, and misuse in the United States. Pain Med.

[CR7] Bornovalova MA, Lejuez CW, Daughters SB, Rosenthal MZ, Lynch TR (2005). Impulsivity as a common process across borderline personality and substance use disorders. Clin Psychol Rev.

[CR8] Burnett S, Bault N, Coricelli G, Blakemore S-J (2010). Adolescents’ heightened riskseeking in a probabilistic gambling task. Cogn Dev.

[CR9] Chen J, Li X, Zhang Q, Zhou Y, Wang R, Tian C, Xiang H (2021). Impulsivity and response inhibition related brain networks in adolescents with internet gaming disorder: a preliminary study utilizing resting-state fMRI. Front Psych.

[CR10] Chong JSX, Ng KK, Tandi J, Wang C, Poh JH, Lo JC, Zhou JH (2019). Longitudinal changes in the cerebral cortex functional organization of healthy elderly. J Neurosci.

[CR11] Ciric R, Wolf DH, Power JD, Roalf DR, Baum GL, Ruparel K, Satterthwaite TD (2017). Benchmarking of participant-level confound regression strategies for the control of motion artifact in studies of functional connectivity. Neuroimage.

[CR12] Coccaro EF, Sripada CS, Yanowitch RN, Phan KL (2011). Corticolimbic function in impulsive aggressive behavior. Biol Psychiat.

[CR13] Dalley JW, Robbins TW (2017). Fractionating impulsivity: neuropsychiatric implications. Nat Rev Neurosci.

[CR14] Dalley JW, Everitt BJ, Robbins TW (2011). Impulsivity, compulsivity, and top-down cognitive control. Neuron.

[CR15] Davis FC, Knodt AR, Sporns O, Lahey BB, Zald DH, Brigidi BD, Hariri AR (2013). Impulsivity and the modular organization of resting-state neural networks. Cereb Cortex.

[CR16] D'Esposito M, Deouell LY, Gazzaley A (2003). Alterations in the BOLD fMRI signal with ageing and disease: a challenge for neuroimaging. Nat Rev Neurosci.

[CR17] Diedenhofen B, Musch J (2015). cocor: A comprehensive solution for the statistical comparison of correlations. PLoS ONE.

[CR18] Doñamayor N, Ebrahimi C, Arndt VA, Weiss F, Schlagenhauf F, Endrass T (2022). Goal-directed and habitual control in human substance use: state of the art and future directions. Neuropsychobiology.

[CR19] Ersche KD, Lim TV, Murley AG, Rua C, Vaghi MM, White TL (2021). Reduced glutamate turnover in the putamen is linked with automatic habits in human cocaine addiction. Biol Psychiatry.

[CR20] Esteban-Penalba T, Paz-Alonso PM, Navalpotro-Gómez I, Rodriguez-Oroz MC (2021). Functional correlates of response inhibition in impulse control disorders in Parkinson’s disease. NeuroImage Clin.

[CR21] Ferrarini L, Veer IM, Baerends E, van Tol MJ, Renken RJ, van der Wee NJ, Milles J (2009). Hierarchical functional modularity in the resting-state human brain. Hum Brain Mapp.

[CR22] Fineberg NA, Chamberlain SR, Goudriaan AE, Stein DJ, Vanderschuren LJMJ, Gillan CM, Potenza MN (2014). New developments in human neurocognition: clinical, genetic, and brain imaging correlates of impulsivity and compulsivity. CNS Spectr.

[CR23] Garon N, Moore C (2006). Negative affectivity predicts individual differences in decision making for preschoolers. J Genet Psychol.

[CR24] Geerligs L, Renken RJ, Saliasi E, Maurits NM, Lorist MM (2015). A brain-wide study of age-related changes in functional connectivity. Cereb Cortex.

[CR25] Gell M, Langner R, Küppers V, Cieslik EC, Satterthwaite TD, Eickhoff SB, Müller VI (2023). Charting the brain networks of impulsivity: Meta-analytic synthesis, functional connectivity modelling and neurotransmitter associations. bioRxiv.

[CR26] Goel V (2019). Hemispheric asymmetry in the prefrontal cortex for complex cognition. Handb Clin Neurol.

[CR27] Grady CL, Garrett DD (2014). Understanding variability in the BOLD signal and why it matters for aging. Brain Imaging Behav.

[CR28] Grafman J, Spector L, Rattermann MJ, Morris R, Wardrafman J (2005). The cognitive psychology of planning, 1st edn.

[CR29] Hammes J, Theis H, Giehl K, Hoenig MC, Greuel A, Tittgemeyer M, van Eimeren T (2019). Dopamine metabolism of the nucleus accumbens and fronto-striatal connectivity modulate impulse control. Brain.

[CR30] Hege MA, Stingl KT, Kullmann S, Schag K, Giel KE, Zipfel S, Preissl H (2015). Attentional impulsivity in binge eating disorder modulates response inhibition performance and frontal brain networks. Int J Obes.

[CR31] Hobkirk AL, Bell RP, Utevsky AV, Huettel S, Meade CS (2019). Reward and executive control network resting-state functional connectivity is associated with impulsivity during reward-based decision making for cocaine users. Drug Alcohol Depend.

[CR32] Inuggi A, Sanz-Arigita E, González-Salinas C, Valero-García AV, García-Santos JM, Fuentes LJ (2014). Brain functional connectivity changes in children that differ in impulsivity temperamental trait. Front Behav Neurosci.

[CR33] Jenkinson M, Bannister P, Brady M, Smith S (2002). Improved optimization for the robust and accurate linear registration and motion correction of brain images. Neuroimage.

[CR34] Jenkinson M, Beckmann CF, Behrens TE, Woolrich MW, Smith SM (2012). Fsl. Neuroimage.

[CR35] Kaller CP, Rahm B, Spreer J, Weiller C, Unterrainer JM (2011). Dissociable contributions of left and right dorsolateral prefrontal cortex in planning. Cereb Cortex.

[CR36] Kämpfe N, Mitte K (2009). A German validation of the UPPS impulsive behavior scale: Further evidence for a four-dimensional model of impulsivity. Eur J Psychol Assess.

[CR37] Kebets V, Favre P, Houenou J, Polosan M, Perroud N, Aubry JM, Piguet C (2021). Fronto-limbic neural variability as a transdiagnostic correlate of emotion dysregulation. Transl Psychiatry.

[CR38] Koh J, Kaneoke Y, Donishi T, Ishida T, Sakata M, Hiwatani Y, Ito H (2020). Increased large-scale inter-network connectivity in relation to impulsivity in Parkinson’s disease. Sci Rep.

[CR39] Kray J, Kreis BK, Lorenz C (2021). Age differences in decision making under known risk: The role of working memory and impulsivity. Dev Psychol.

[CR40] Kumar U, Arya A, Agarwal V (2022). Altered functional connectivity in children with ADHD while performing cognitive control task. Psychiatry Res Neuroimaging.

[CR41] Latvala T, Lintonen T, Konu A (2019). Public health effects of gambling–debate on a conceptual model. BMC Public Health.

[CR42] Lee SW, Shimojo S, O’Doherty JP (2014). Neural computations underlying arbitration between model-based and model-free learning. Neuron.

[CR43] Lee RS, Hoppenbrouwers S, Franken I (2019). A systematic meta-review of impulsivity and compulsivity in addictive behaviors. Neuropsychol Rev.

[CR44] Leijenhorst LV, Westenberg PM, Crone EA (2008). A developmental study of risky decisions on the Cake Gambling Task: age and gender analyses of probability estimation and reward evaluation. Dev Neuropsychol.

[CR45] Levin KA (2006). Study design III: Cross-sectional studies. Evid Based Dent.

[CR46] Liu P, Feng T (2017). The overlapping brain region accounting for the relationship between procrastination and impulsivity: a voxel-based morphometry study. Neuroscience.

[CR47] Lydon-Staley DM, Ciric R, Satterthwaite TD, Bassett DS (2019). Evaluation of confound regression strategies for the mitigation of micromovement artifact in studies of dynamic resting-state functional connectivity and multilayer network modularity. Netw Neurosci.

[CR48] Manthey J, Hassan SA, Carr S, Kilian C, Kuitunen-Paul S, Rehm J (2021). What are the economic costs to society attributable to alcohol use? A systematic review and modelling study. Pharmacoeconomics.

[CR49] Maxwell AL, Gardiner E, Loxton NJ (2020). Investigating the relationship between reward sensitivity, impulsivity, and food addiction: a systematic review. Eur Eat Disord Rev.

[CR50] Meunier D, Achard S, Morcom A, Bullmore E (2009). Age-related changes in modular organization of human brain functional networks. Neuroimage.

[CR51] Middleton FA, Strick PL (2000). Basal ganglia output and cognition: evidence from anatomical, behavioral, and clinical studies. Brain Cogn.

[CR52] Miller AE, Racine SE (2022). Emotion regulation difficulties as common and unique predictors of impulsive behaviors in university students. J Am Coll Health.

[CR53] Moeller FG, Barratt ES, Dougherty DM, Schmitz JM, Swann AC (2001). Psychiatric aspects of impulsivity. Am J Psychiatry.

[CR54] Molina-Ruiz RM, García-Saiz T, Looi JC, Virgili EV, Zamorano MR, de Anta Tejado L, Díaz-Marsá M (2020). Neural mechanisms in eating behaviors: a pilot fMRI study of emotional processing. Psychiatry Investig.

[CR55] Morein-Zamir S, Robbins TW (2015). Fronto-striatal circuits in response-inhibition: Relevance to addiction. Brain Res.

[CR56] Motzkin JC, Baskin-Sommers A, Newman JP, Kiehl KA, Koenigs M (2014). Neural correlates of substance abuse: reduced functional connectivity between areas underlying reward and cognitive control. Hum Brain Mapp.

[CR57] Oliva R, Morys F, Horstmann A, Castiello U, Begliomini C (2020). Characterizing impulsivity and resting-state functional connectivity in normal-weight binge eaters. Int J Eat Disord.

[CR58] Olson IR, Plotzker A, Ezzyat Y (2007). The enigmatic temporal pole: a review of findings on social and emotional processing. Brain.

[CR59] Pan N, Wang S, Zhao Y, Lai H, Qin K, Li J, Gong Q (2021). Brain gray matter structures associated with trait impulsivity: a systematic review and voxel-based meta-analysis. Hum Brain Mapp.

[CR60] Parkes L, Fulcher B, Yucel M, Fornito A (2018). An evaluation of the efficacy, reliability, and sensitivity of motion correction strategies for resting-state functional MRI. Neuroimage.

[CR61] Paulsen D, Carter RM, Platt M, Huettel SA, Brannon EM (2012). Neurocognitive development of risk aversion from early childhood to adulthood. Front Hum Neurosci.

[CR62] Pruessner L, Barnow S, Holt DV, Joormann J, Schulze K (2020). A cognitive control framework for understanding emotion regulation flexibility. Emotion.

[CR63] Puxeddu MG, Faskowitz J, Betzel RF, Petti M, Astolfi L, Sporns O (2020). The modular organization of brain cortical connectivity across the human lifespan. Neuroimage.

[CR64] Quaglieri A, Mari E, Boccia M, Piccardi L, Guariglia C, Giannini AM (2020). Brain network underlying executive functions in gambling and alcohol use disorders: an activation likelihood estimation meta-analysis of fMRI studies. Brain Sci.

[CR65] Ravaja N, Somervuori O, Salminen M (2013). Predicting purchase decision: the role of hemispheric asymmetry over the frontal cortex. J Neurosci Psychol Econ.

[CR66] Reynolds BW, Basso MR, Miller AK, Whiteside DM, Combs D (2019). Executive function, impulsivity, and risky behaviors in young adults. Neuropsychology.

[CR67] Schaefer A, Kong R, Gordon EM, Laumann TO, Zuo XN, Holmes AJ, Yeo BT (2018). Local-global parcellation of the human cerebral cortex from intrinsic functional connectivity MRI. Cereb Cortex.

[CR68] Schilling C, Kühn S, Romanowski A, Banaschewski T, Barbot A, Barker GJ, IMAGEN consortium (2013). Common structural correlates of trait impulsiveness and perceptual reasoning in adolescence. Hum Brain Mapp.

[CR69] Smith SM (2002). Fast robust automated brain extraction. Hum Brain Mapp.

[CR70] Smith SM, Jenkinson M, Woolrich MW, Beckmann CF, Behrens TE, Johansen-Berg H, Matthews PM (2004). Advances in functional and structural MR image analysis and implementation as FSL. Neuroimage.

[CR71] Soltani A, Koechlin E (2022). Computational models of adaptive behavior and prefrontal cortex. Neuropsychopharmacology.

[CR72] Song J, Birn RM, Boly M, Meier TB, Nair VA, Meyerand ME, Prabhakaran V (2014). Age-related reorganizational changes in modularity and functional connectivity of human brain networks. Brain Connectivity.

[CR73] Tessitore A, Santangelo G, De Micco R, Giordano A, Raimo S, Amboni M, Vitale C (2017). Resting-state brain networks in patients with Parkinson’s disease and impulse control disorders. Cortex.

[CR74] Tian Y, Margulies DS, Breakspear M, Zalesky A (2020). Topographic organization of the human subcortex unveiled with functional connectivity gradients. Nat Neurosci.

[CR75] Vaghi MM, Vértes PE, Kitzbichler MG, Apergis-Schoute AM, van der Flier FE, Fineberg NA, Robbins TW (2017). Specific frontostriatal circuits for impaired cognitive flexibility and goal-directed planning in obsessive-compulsive disorder: evidence from resting-state functional connectivity. Biol Psychiat.

[CR76] Vallesi A (2021). The quest for hemispheric asymmetries supporting and predicting executive functioning. J Cogn Neurosci.

[CR77] van den Heuvel MP, de Lange SC, Zalesky A, Seguin C, Yeo BT, Schmidt R (2017). Proportional thresholding in resting-state fMRI functional connectivity networks and consequences for patient-control connectome studies: Issues and recommendations. Neuroimage.

[CR78] Van Overwalle F, Baetens K (2009). Understanding others' actions and goals by mirror and mentalizing systems: a meta-analysis. Neuroimage.

[CR79] Van Wijk BC, Stam CJ, Daffertshofer A (2010). Comparing brain networks of different size and connectivity density using graph theory. PLoS ONE.

[CR80] Wang J, Fan Y, Dong Y, Ma M, Ma Y, Dong Y, Cui C (2016). Alterations in brain structure and functional connectivity in alcohol dependent patients and possible association with impulsivity. PLoS ONE.

[CR81] Whelan R, Conrod PJ, Poline JB, Lourdusamy A, Banaschewski T, Barker GJ, Imagen Consortium (2012). Adolescent impulsivity phenotypes characterized by distinct brain networks. Nat Neurosci.

[CR82] Whiteside SP, Lynam DR (2001). The five factor model and impulsivity: Using a structural model of personality to understand impulsivity. Pers Individ Differ.

[CR83] Whitfield-Gabrieli S, Nieto-Castanon A (2012). Conn: a functional connectivity toolbox for correlated and anticorrelated brain networks. Brain Connect.

[CR84] Wilcox CE, Abbott CC, Calhoun VD (2019). Alterations in resting-state functional connectivity in substance use disorders and treatment implications. Prog Neuropsychopharmacol Biol Psychiatry.

[CR85] Xu T, Gu Q, Zhao Q, Wang P, Liu Q, Fan Q, Wang Z (2021). Impaired cortico-striatal functional connectivity is related to trait impulsivity in unmedicated patients with obsessive-compulsive disorder. J Affect Disord.

[CR100] Yeo BT, Ktienen FM, Sepulcre J (2011). Aberrant functional connectivity in resting state networks of ADHD patients revealed by independent component analysis. BMC Neurosci.

[CR86] Zhang H, Zhao Y, Cao W, Cui D, Jiao Q, Lu W, Qiu J (2020). Aberrant functional connectivity in resting state networks of ADHD patients revealed by independent component analysis. BMC Neurosci.

[CR87] Zhao Q, Sang X, Metmer H, Lu J, Initiative ADN (2019). Functional segregation of executive control network and frontoparietal network in Alzheimer's disease. Cortex.

