# Supplementary material for: Topological features of functional brain networks and subclinical impulsivity: an investigation in younger and older adults
Source: Brain Struct Funct. 2024 Mar 6;229(4):865–77. doi: 10.1007/s00429-023-02745-5 (PMC11003924; doi:10.1007/s00429-023-02745-5)
Supplement: Supplementary file 1 — Supplementary file1 (DOCX 524 KB) [file 429_2023_2745_MOESM1_ESM.docx]

# Supplementary materials

|  |  |  | **MNI coordinates** | | |
| --- | --- | --- | --- | --- | --- |
| **Atlas** | **Network** | **Label (as per atlas)** | **x** | **y** | **z** |
| Schaefer | Frontal | LH_Cont_OFC_1 | -32 | 42 | -14 |
| Schaefer | Frontal | LH_Cont_PFCl_1 | -42 | 50 | -6 |
| Schaefer | Frontal | LH_Cont_PFCl_2 | -28 | 58 | 8 |
| Schaefer | Frontal | LH_Cont_PFCl_3 | -42 | 40 | 16 |
| Schaefer | Frontal | LH_Cont_PFCl_4 | -44 | 20 | 28 |
| Schaefer | Frontal | LH_Cont_PFCl_5 | -42 | 6 | 44 |
| Schaefer | Frontal | RH_Cont_PFCv_1 | 34 | 22 | -8 |
| Schaefer | Frontal | RH_Cont_PFCl_1 | 36 | 46 | -14 |
| Schaefer | Frontal | RH_Cont_PFCl_2 | 30 | 58 | 4 |
| Schaefer | Frontal | RH_Cont_PFCl_3 | 44 | 44 | 10 |
| Schaefer | Frontal | RH_Cont_PFCl_4 | 46 | 24 | 26 |
| Schaefer | Frontal | RH_Cont_PFCl_5 | 30 | 48 | 28 |
| Schaefer | Frontal | RH_Cont_PFCl_6 | 40 | 34 | 38 |
| Schaefer | Frontal | RH_Cont_PFCl_7 | 42 | 14 | 48 |
| Schaefer | Limbic | LH_Limbic_OFC_1 | -24 | 22 | -20 |
| Schaefer | Limbic | LH_Limbic_OFC_2 | -10 | 36 | -20 |
| Schaefer | Limbic | LH_Limbic_TempPole_1 | -30 | -6 | -40 |
| Schaefer | Limbic | LH_Limbic_TempPole_2 | -44 | -20 | -30 |
| Schaefer | Limbic | LH_Limbic_TempPole_3 | -28 | 10 | -34 |
| Schaefer | Limbic | LH_Limbic_TempPole_4 | -42 | 8 | -18 |
| Schaefer | Limbic | RH_Limbic_OFC_1 | 12 | 38 | -22 |
| Schaefer | Limbic | RH_Limbic_OFC_2 | 28 | 22 | -20 |
| Schaefer | Limbic | RH_Limbic_OFC_3 | 14 | 64 | -8 |
| Schaefer | Limbic | RH_Limbic_TempPole_1 | 30 | 8 | -38 |
| Schaefer | Limbic | RH_Limbic_TempPole_2 | 46 | -12 | -34 |
| Schaefer | Limbic | RH_Limbic_TempPole_3 | 26 | -10 | -32 |
| Tian | Limbic | HIP-RH | 27.190769 | -22.18154 | -14.13846 |
| Tian | Limbic | AMY-RH | 24.165714 | -3.748571 | -18.66286 |
| Tian | Limbic | HIP-LH | -25.19077 | -22.18154 | -14.13846 |
| Tian | Limbic | AMY-LH | -22.16571 | -3.748571 | -18.66286 |
| Tian | Striatal | Nac-RH | 12.737864 | 14.271845 | -5.106796 |
| Tian | Striatal | GP-RH | 20.121212 | -3.818182 | -1.69697 |
| Tian | Striatal | PUT-RH | 26.786192 | 0.44098 | 0.835189 |
| Tian | Striatal | CAU-RH | 13.917241 | 10.726437 | 10.009195 |
| Tian | Striatal | Nac-LH | -10.73786 | 14.271845 | -5.106796 |
| Tian | Striatal | GP-LH | -18.12121 | -3.818182 | -1.69697 |
| Tian | Striatal | PUT-LH | -24.78619 | 0.44098 | 0.835189 |
| Tian | Striatal | CAU-LH | -11.91724 | 10.726437 | 10.009195 |

Table S1 - List of cortical and subcortical a priori selected ROIs. AMY: amygdala; CAU: caudate; HIP: hippocampus; GP: globus pallidus; LH: left hemisphere; Nac: nucleus accumbens; OFC: orbitofrontal cortex; PFCl: lateral prefrontal cortex; PFCv: ventral prefrontal cortex; PUT: putamen; RH: right hemisphere. TempPole: temporal pole.

| *Frontal network* | | | | | |
| --- | --- | --- | --- | --- | --- |
|  | ClusCoef | Deg | Eccen | Eloc | PartCoef |
| ClusCoef | 1 | 0.465996527 | -0.46355995 | 0.612175874 | 0.408801028 |
| Deg | 0.465996527 | 1 | -0.28232517 | 0.730422168 | 0.831274446 |
| Eccen | -0.46355995 | -0.28232517 | 1 | -0.083676 | -0.2998985 |
| Eloc | 0.612175874 | 0.730422168 | -0.083676 | 1 | 0.616931822 |
| PartCoef | 0.408801028 | 0.831274446 | -0.2998985 | 0.616931822 | 1 |
| *Limbic network* | | | | | |
|  | ClusCoef | Deg | Eccen | Eloc | PartCoef |
| ClusCoef | 1 | 0.555729681 | -0.17038226 | 0.755699268 | 0.479465219 |
| Deg | 0.555729681 | 1 | 0.048292517 | 0.808950002 | 0.782572482 |
| Eccen | -0.17038226 | 0.048292517 | 1 | 0.181215901 | 0.376328593 |
| Eloc | 0.755699268 | 0.808950002 | 0.181215901 | 1 | 0.799984242 |
| PartCoef | 0.479465219 | 0.782572482 | 0.376328593 | 0.799984242 | 1 |
| *Striatal network* | | | | | |
|  | ClusCoef | Deg | Eccen | Eloc | PartCoef |
| ClusCoef | 1 | 0.282712783 | -0.04568834 | 0.674378373 | 0.394723348 |
| Deg | 0.282712783 | 1 | -0.11067637 | 0.494407127 | 0.842669839 |
| Eccen | -0.04568834 | -0.11067637 | 1 | 0.316309137 | 0.088726194 |
| Eloc | 0.674378373 | 0.494407127 | 0.316309137 | 1 | 0.72991014 |
| PartCoef | 0.394723348 | 0.842669839 | 0.088726194 | 0.72991014 | 1 |

Table S2 - Correlations (rho values) between mean graph-theoretical network metrics. ClusCoef: clustering coefficient; Deg: degree; Eccen: eccentricity; Eloc: local efficiency; PartCoef: participation coefficient

## Testing robustness of main results with different thresholding methods

Robustness of significant results reported in the main text (obtained by keeping the 20% strongest connections) was investigated by repeating all statistical analyses leading to significant results after thresholding connectivity matrices while preserving the 10%, 30% and 40% strongest connections ( for both younger and older individuals).

### Younger 10%


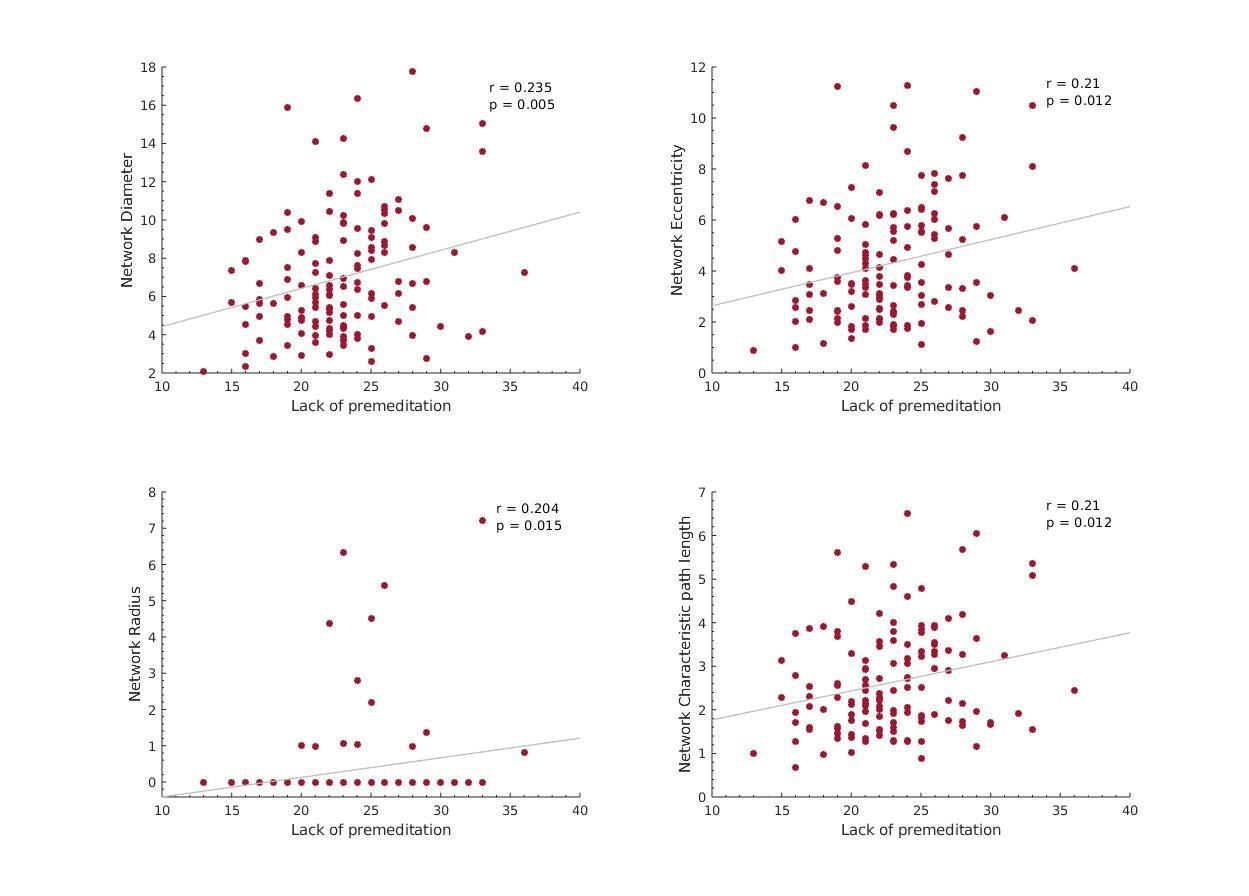


Figure S1 - Significant correlations between network measures (all nodes considered together) and UPPS scores for younger individuals (threshold: 10%)


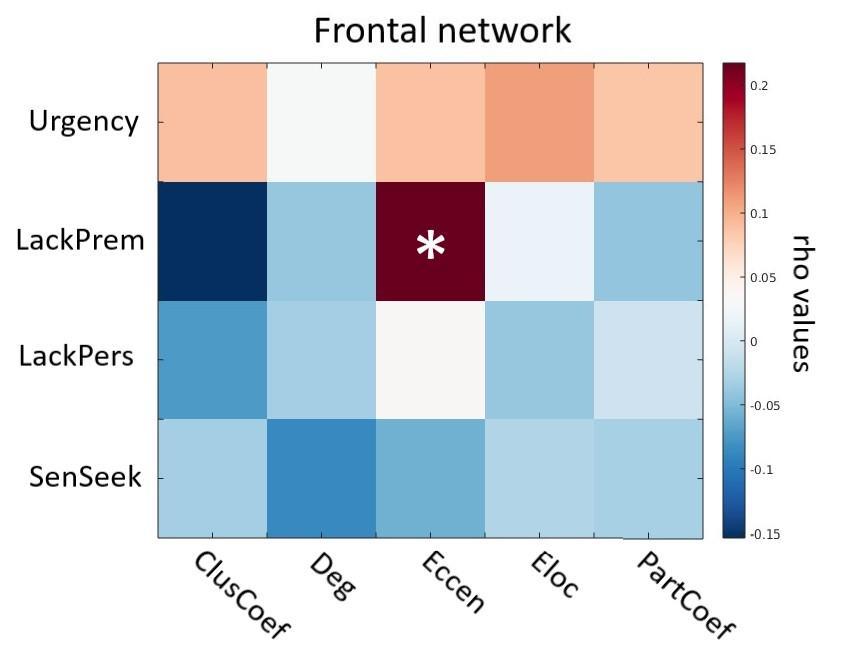


Figure S2 - Correlations between network measures (i.e., mean nodal measures computed separately for frontal, limbic and striatal components) and UPPS scores for younger individuals (threshold: 10%). ClusCoef: clustering coefficient; Deg: degree; Eccen: eccentricity; Eloc: local efficiency; LackPers: lack of perseverance; LackPrem: lack of premeditation; PartCoef: participation coefficient; SenSeek: sensation.seeking


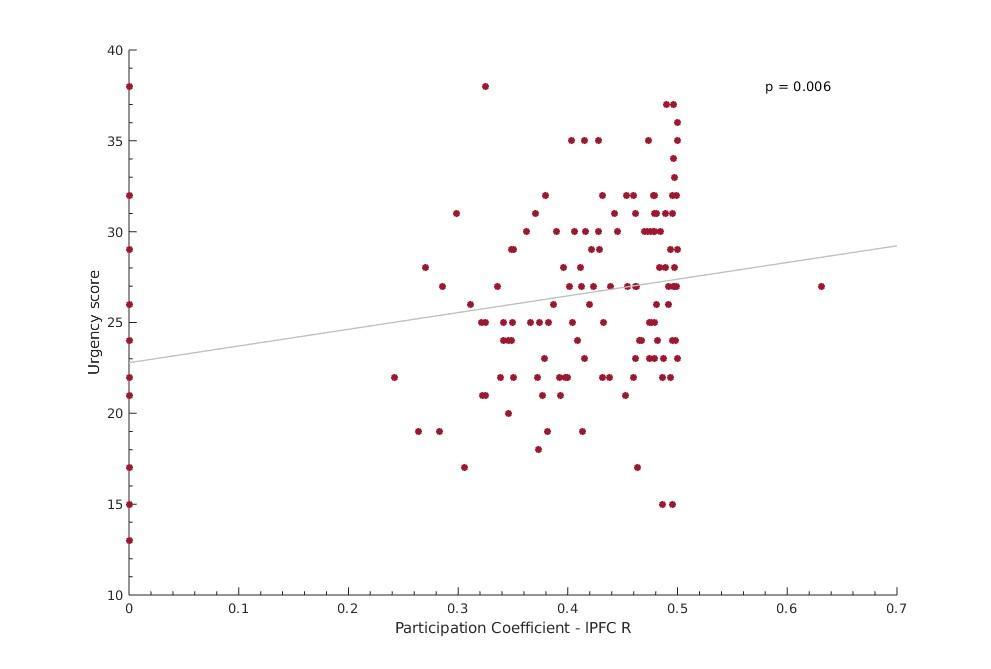


Figure S3 - Significant correlations between nodal measures (i.e., metrics separately computed for each node) and UPPS scores for younger individuals (threshold: 10%). lPFC R: right lateral prefrontal cortex

| *Lack of premeditation ~ Frontal metrics* | | | | | | | |
| --- | --- | --- | --- | --- | --- | --- | --- |
|  | Estimate | SE | tStat | DF | pValue | Lower | Upper |
| (Intercept) | 20.534 | 1.4695 | 13.974 | 133 | 7.19E-28 | 17.628 | 23.441 |
| ClusCoef frontal | -4.8363 | 3.5297 | -1.3702 | 133 | 0.17295 | -11.818 | 2.1454 |
| Deg frontal | -0.27062 | 0.30666 | -0.88246 | 133 | 0.37912 | -0.87718 | 0.33595 |
| **Eccen frontal*** | 0.32972 | 0.14005 | 2.3544 | 133 | 0.020018 | 0.052715 | 0.60673 |
| Eloc frontal | 5.3909 | 4.5974 | 1.1726 | 133 | 0.24306 | -3.7026 | 14.484 |
| PartCoef frontal | 3.6196 | 5.4601 | 0.66292 | 133 | 0.50853 | -7.1802 | 14.419 |
|  | | | | | | | |
| *Lack of premeditation ~ Limbic metrics* | | | | | | | |
|  | Estimate | SE | tStat | DF | pValue | Lower | Upper |
| (Intercept) | 20.895 | 0.77374 | 27.005 | 132 | 1.31E-55 | 19.364 | 22.425 |
| ClusCoef limbic | 2.9016 | 5.525 | 0.52517 | 132 | 0.60035 | -8.0275 | 13.831 |
| Deg limbic | 0.33508 | 0.56362 | 0.59452 | 132 | 0.55318 | -0.77981 | 1.45 |
| **Eccent limbic*** | 0.66987 | 0.20051 | 3.3408 | 132 | 0.001087 | 0.27324 | 1.0665 |
| Eloc limbic | 0.61959 | 5.9871 | 0.10349 | 132 | 0.91773 | -11.224 | 12.463 |
| PartCoef limbic | -10.304 | 6.1682 | -1.6706 | 132 | 0.097178 | -22.506 | 1.897 |
|  | | | | | | | |
| *Sensation-seeking ~ Limbic metrics* | | | | | | | |
|  | Estimate | SE | tStat | DF | pValue | Lower | Upper |
| (Intercept) | 34.479 | 1.381 | 24.968 | 136 | 1.22E-52 | 31.748 | 37.21 |
| ClusCoef limbic | 6.8338 | 9.8838 | 0.69142 | 136 | 0.49048 | -12.712 | 26.38 |
| **Deg limbic*** | 2.3134 | 0.99589 | 2.3229 | 136 | 0.021667 | 0.34395 | 4.2828 |
| Eccen limbic | 0.077474 | 0.3567 | 0.21719 | 136 | 0.82838 | -0.62793 | 0.78288 |
| Eloc limbic | -18.752 | 10.735 | -1.7469 | 136 | 0.082917 | -39.98 | 2.4764 |
| PartCoef limbic | -7.9141 | 10.769 | -0.73489 | 136 | 0.46367 | -29.211 | 13.383 |

Table S3 – Significant Linear Mixed-effect Models (LMMs) predicting UPPS subscale scores from mean network metrics (i.e., ClusCoef: clustering coefficient; Deg: degree; Eccen: eccentricity; Eloc: local efficiency; PartCoef: participation coefficient) in younger individuals, divided according to frontal, limbic and striatal components (threshold: 10%)

### Younger 30%


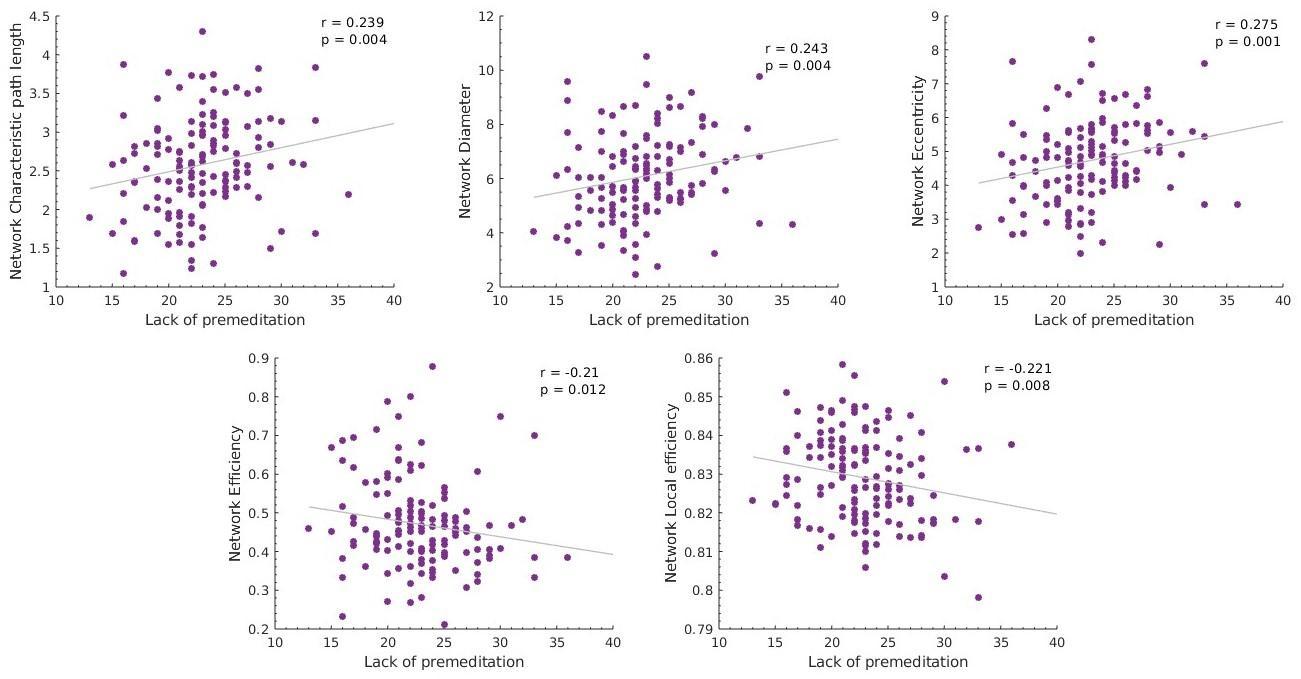


Figure S4 - Significant correlations between network measures (all nodes considered together) and UPPS scores for younger individuals (threshold: 30%)


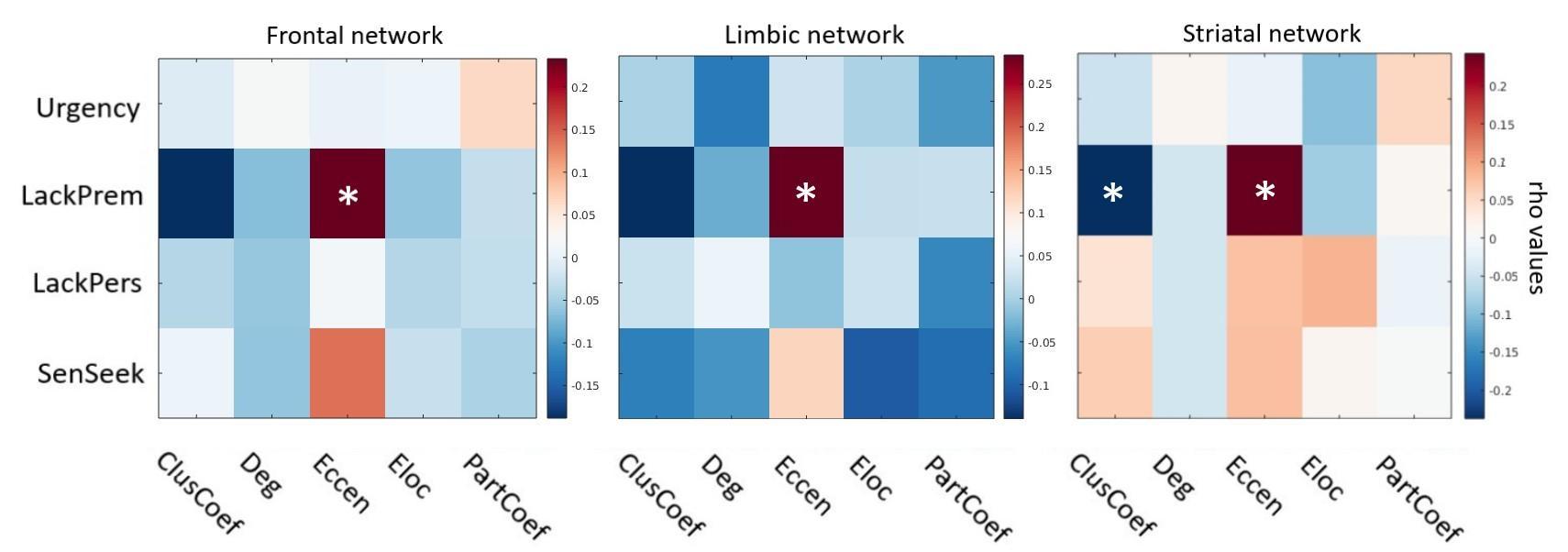


Figure S5 - Correlations between network measures (i.e., mean nodal measures computed separately for frontal, limbic and striatal components) and UPPS scores for younger individuals (threshold: 30%). ClusCoef: clustering coefficient; Deg: degree; Eccen: eccentricity; Eloc: local efficiency; LackPers: lack of perseverance; LackPrem: lack of premeditation; PartCoef: participation coefficient; SenSeek: sensation.seeking


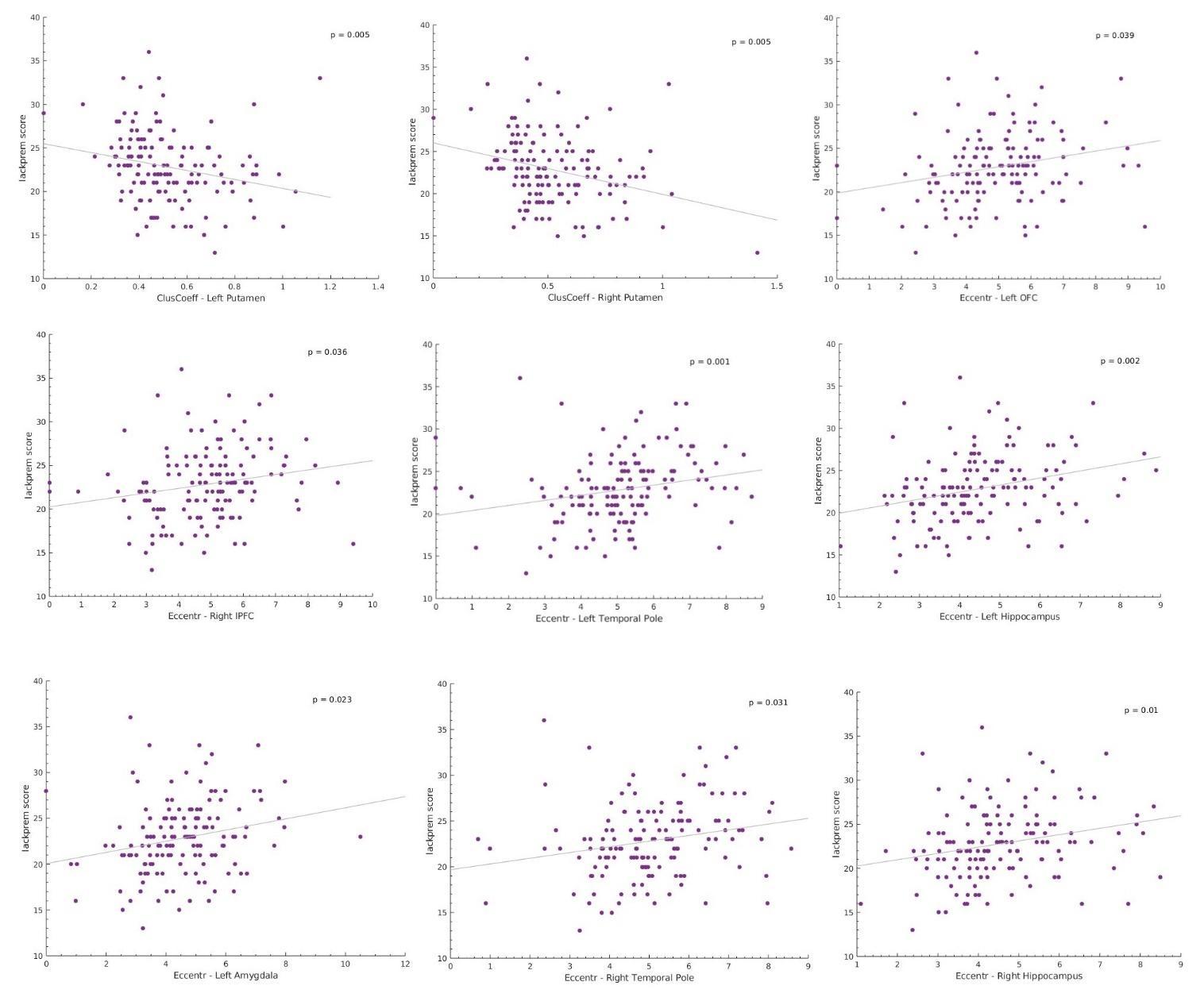


Figure S6 – Significant correlations between nodal measures (i.e., metrics separately computed for each node) and UPPS scores for younger individuals (threshold: 30%). Eccentr: eccentricity; lPFC: lateral prefrontal cortex; lackprem: lack of premeditation; OFC: orbitofrontal cortex

| *Urgency ~ Frontal metrics* | | | | | | | |
| --- | --- | --- | --- | --- | --- | --- | --- |
|  | Estimate | SE | tStat | DF | pValue | Lower | Upper |
| (Intercept) | 22.007 | 7.2288 | 3.0443 | 136 | 0.0028006 | 7.7113 | 36.302 |
| ClusCoef frontal | 1.847 | 3.6242 | 0.50963 | 136 | 0.61114 | -5.32 | 9.014 |
| Deg frontal | -0.37818 | 0.23046 | -1.641 | 136 | 0.10311 | -0.83393 | 0.077568 |
| Eccen frontal | 0.19106 | 0.43355 | 0.44068 | 136 | 0.66014 | -0.66631 | 1.0484 |
| Eloc frontal | -6.4501 | 9.9856 | -0.64594 | 136 | 0.51941 | -26.197 | 13.297 |
| **PartCoef frontal*** | 24.844 | 10.619 | 2.3395 | 136 | 0.020763 | 3.8439 | 45.844 |
|  | | | | | | | |
| *Lack of premeditation ~ Frontal metrics* | | | | | | | |
|  | Estimate | SE | tStat | DF | pValue | Lower | Upper |
| (Intercept) | 11.199 | 5.6154 | 1.9943 | 135 | 0.048135 | 0.093178 | 22.304 |
| ClusCoef frontal | -0.91562 | 2.8485 | -0.32144 | 135 | 0.74837 | -6.549 | 4.7178 |
| Deg frontal | -0.25392 | 0.17895 | -1.419 | 135 | 0.15821 | -0.60783 | 0.099981 |
| **Eccen frontal*** | 0.78103 | 0.33981 | 2.2984 | 135 | 0.023073 | 0.10899 | 1.4531 |
| Eloc frontal | 5.7896 | 7.7377 | 0.74823 | 135 | 0.45562 | -9.5133 | 21.092 |
| PartCoef frontal | 13.708 | 8.2664 | 1.6582 | 135 | 0.099593 | -2.6408 | 30.056 |
|  | | | | | | | |
| *Lack of premeditation ~ Limbic metrics* | | | | | | | |
|  | Estimate | SE | tStat | DF | pValue | Lower | Upper |
| (Intercept) | 18.314 | 2.9294 | 6.2517 | 134 | 5.05E-09 | 12.52 | 24.108 |
| ClusCoef limbic | -1.2241 | 3.6094 | -0.33913 | 134 | 0.73504 | -8.3628 | 5.9146 |
| Deg limbic | -0.049646 | 0.19481 | -0.25485 | 134 | 0.79923 | -0.43494 | 0.33565 |
| **Eccen limbic*** | 0.85821 | 0.33176 | 2.5869 | 134 | 0.010752 | 0.20205 | 1.5144 |
| Eloc limbic | 5.18 | 5.8286 | 0.88873 | 134 | 0.37574 | -6.3479 | 16.708 |
| PartCoef limbic | -4.3947 | 8.2409 | -0.53328 | 134 | 0.59472 | -20.694 | 11.904 |
|  | | | | | | | |
| *Lack of perseverance ~ Limbic metrics* | | | | | | | |
|  | Estimate | SE | tStat | DF | pValue | Lower | Upper |
| (Intercept) | 24 | 3.7818 | 6.3463 | 136 | 3.05E-09 | 16.522 | 31.479 |
| ClusCoef limbic | -2.8093 | 4.6525 | -0.60382 | 136 | 0.54697 | -12.01 | 6.3914 |
| Deg limbic | 0.24315 | 0.25124 | 0.96781 | 136 | 0.33486 | -0.25369 | 0.73998 |
| Eccen limbic | 0.3241 | 0.42636 | 0.76015 | 136 | 0.44848 | -0.51906 | 1.1673 |
| Eloc limbic | 12.392 | 7.4299 | 1.6679 | 136 | 0.097633 | -2.3006 | 27.085 |
| **PartCoef limbic*** | -29.127 | 10.711 | -2.7193 | 136 | 0.0073957 | -50.31 | -7.945 |
|  | | | | | | | |
| *Sensation-seeking ~ Limbic metrics* | | | | | | | |
|  | Estimate | SE | tStat | DF | pValue | Lower | Upper |
| (Intercept) | 27.393 | 4.7561 | 5.7594 | 135 | 5.44E-08 | 17.986 | 36.799 |
| ClusCoef limbic | 11.474 | 5.8482 | 1.9619 | 135 | 0.05183 | -0.092295 | 23.04 |
| Deg limbic | 0.5264 | 0.31606 | 1.6655 | 135 | 0.098135 | -0.098677 | 1.1515 |
| **Eccen limbic*** | 1.4196 | 0.53607 | 2.6482 | 135 | 0.009055 | 0.35946 | 2.4798 |
| **Eloc limbic*** | -23.749 | 9.3381 | -2.5432 | 135 | 0.012111 | -42.217 | -5.281 |
| PartCoef limbic | 14.572 | 13.473 | 1.0815 | 135 | 0.2814 | -12.075 | 41.218 |
|  | | | | | | | |
| *Lack of premeditation ~ Striatal metrics* | | | | | | | |
|  | Estimate | SE | tStat | DF | pValue | Lower | Upper |
| (Intercept) | 21.339 | 2.5742 | 8.2895 | 134 | 1.05E-13 | 16.247 | 26.43 |
| ClusCoef striatal | -2.4614 | 2.604 | -0.94523 | 134 | 0.34624 | -7.6117 | 2.6889 |
| Deg striatal | -0.065633 | 0.11119 | -0.59026 | 134 | 0.55601 | -0.28556 | 0.15429 |
| **Eccen striatal*** | 0.80723 | 0.29276 | 2.7574 | 134 | 0.0066405 | 0.22821 | 1.3862 |
| Eloc striatal | -6.9339 | 4.5255 | -1.5322 | 134 | 0.12783 | -15.885 | 2.0168 |
| PartCoef striatal | 10.122 | 6.6904 | 1.5129 | 134 | 0.13266 | -3.1105 | 23.354 |
|  | | | | | | | |
| *Lack of perseverance ~ Striatal metrics* | | | | | | | |
|  | Estimate | SE | tStat | DF | pValue | Lower | Upper |
| (Intercept) | 19.791 | 3.3787 | 5.8575 | 135 | 3.41E-08 | 13.109 | 26.473 |
| ClusCoef striatal | 4.4693 | 3.3529 | 1.3329 | 135 | 0.1848 | -2.1618 | 11.1 |
| Deg striatal | 0.18904 | 0.14638 | 1.2914 | 135 | 0.19877 | -0.10046 | 0.47853 |
| **Eccen striatal*** | 0.75933 | 0.38174 | 1.9891 | 135 | 0.048709 | 0.0043632 | 1.5143 |
| Eloc striatal | -3.7435 | 5.9338 | -0.63088 | 135 | 0.52919 | -15.479 | 7.9917 |
| PartCoef striatal | -9.5424 | 8.7892 | -1.0857 | 135 | 0.27955 | -26.925 | 7.8399 |

Table S4 - Significant Linear Mixed-effect Models (LMMs) predicting UPPS subscale scores from mean network metrics (i.e., ClusCoef: clustering coefficient; Deg: degree; Eccen: eccentricity; Eloc: local efficiency; PartCoef: participation coefficient) in younger individuals, divided according to frontal, limbic and striatal components (threshold: 30%)

### Young 40%


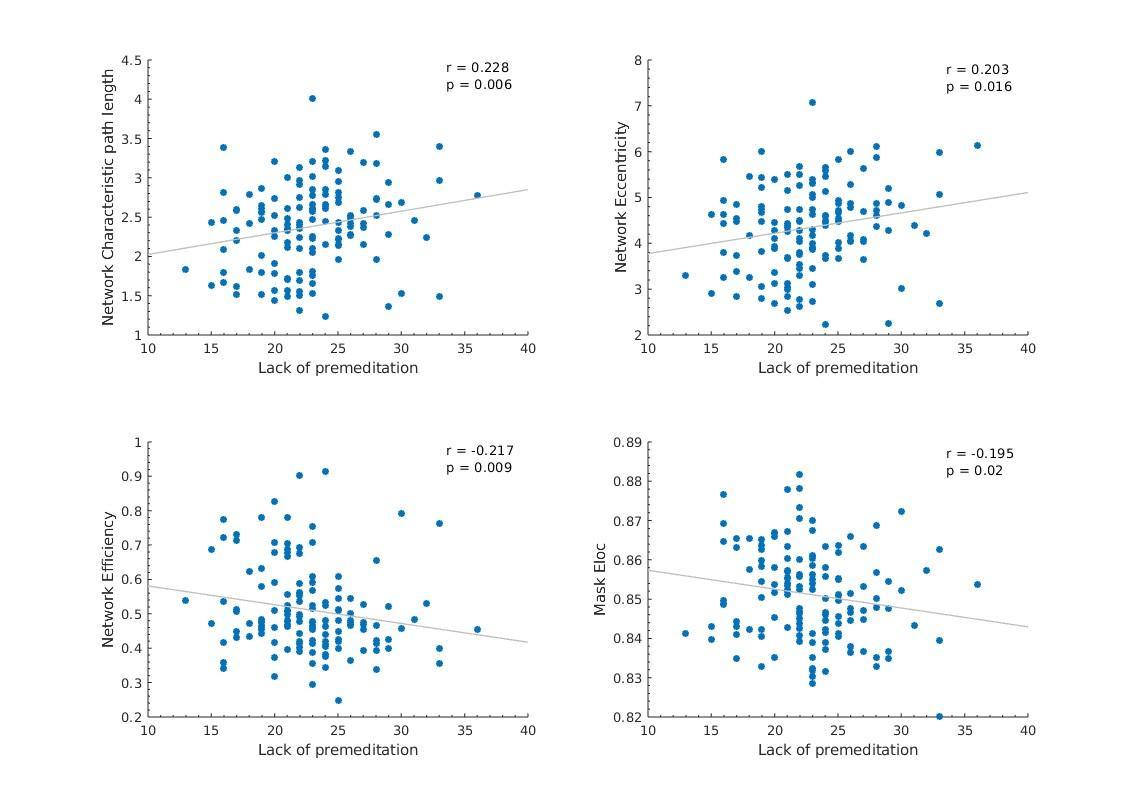


Figure S7 - Significant correlations between network measures (all nodes considered together) and UPPS scores for younger individuals (threshold: 40%)


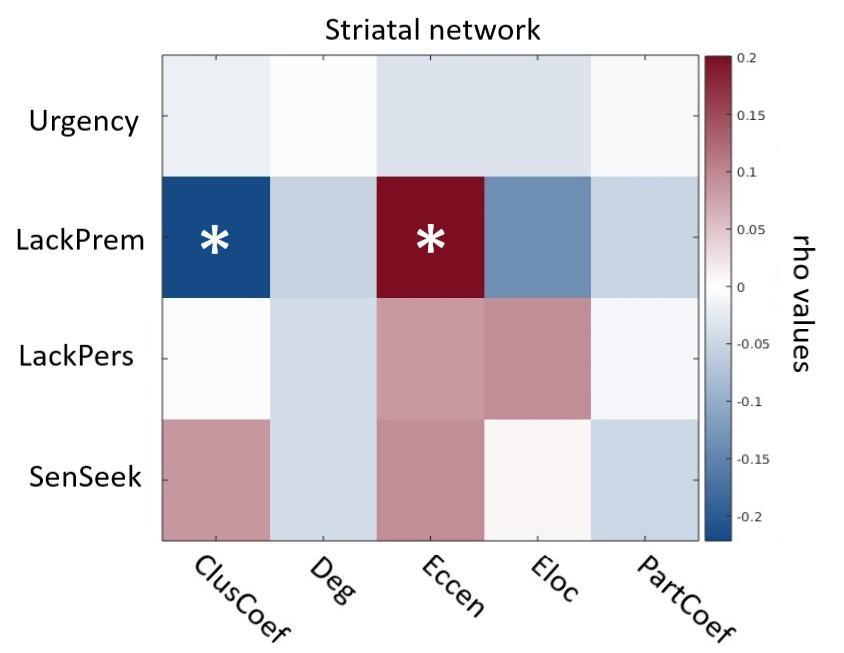


Figure S8 - Correlations between network measures (i.e., mean nodal measures computed separately for frontal, limbic and striatal components) and UPPS scores for younger individuals (threshold: 40%). ClusCoef: clustering coefficient; Deg: degree; Eccen: eccentricity; Eloc: local efficiency; LackPers: lack of perseverance; LackPrem: lack of premeditation; PartCoef: participation coefficient; SenSeek: sensation.seeking


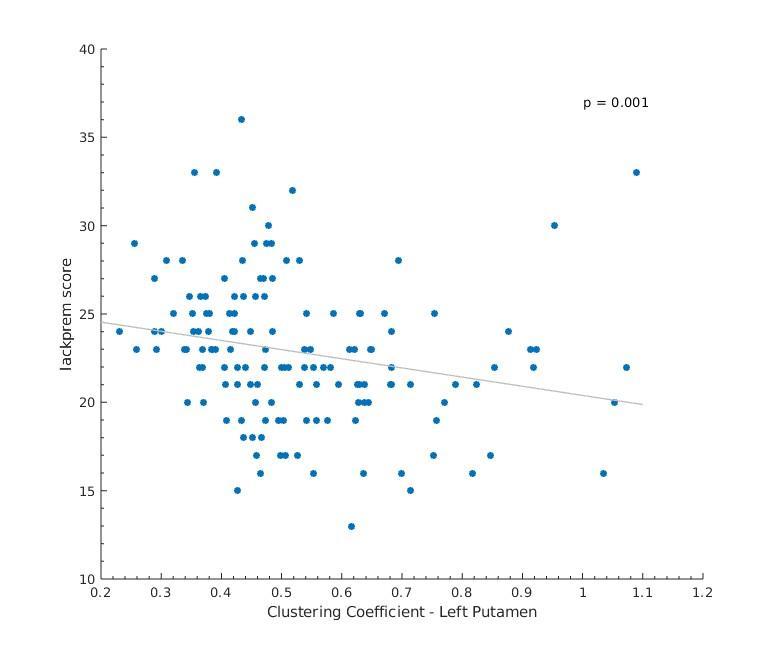


Figure S9 - Significant correlations between nodal measures (i.e., metrics separately computed for each node) and UPPS scores for younger individuals (threshold: 40%). lackprem: lack of premeditation

| *Urgency ~ Frontal metrics* | | | | | | | |
| --- | --- | --- | --- | --- | --- | --- | --- |
|  | Estimate | SE | tStat | DF | pValue | Lower | Upper |
| (Intercept) | 10.536 | 11.482 | 0.91762 | 136 | 0.36044 | -12.171 | 33.243 |
| ClusCoef frontal | 0.48675 | 3.856 | 0.12623 | 136 | 0.89973 | -7.1387 | 8.1121 |
| **Deg frontal*** | -0.51129 | 0.25265 | -2.0237 | 136 | 0.044959 | -1.0109 | -0.01166 |
| Eccen frontal | -0.00943 | 0.59397 | -0.015875 | 136 | 0.98736 | -1.184 | 1.1652 |
| Eloc frontal | 10.766 | 15.228 | 0.70702 | 136 | 0.48077 | -19.348 | 40.88 |
| **PartCoef frontal*** | 27.238 | 12.984 | 2.0977 | 136 | 0.037781 | 1.5602 | 52.915 |
|  | | | | | | | |
| *Lack of premeditation ~ Frontal metrics* | | | | | | | |
|  | Estimate | SE | tStat | DF | pValue | Lower | Upper |
| (Intercept) | 5.329 | 8.9271 | 0.59695 | 135 | 0.55154 | -12.326 | 22.984 |
| ClusCoef frontal | -4.2618 | 3.0329 | -1.4052 | 135 | 0.16226 | -10.26 | 1.7363 |
| **Deg frontal*** | -0.43738 | 0.19642 | -2.2268 | 135 | 0.027618 | -0.82584 | -0.04893 |
| Eccen frontal | 0.55888 | 0.46173 | 1.2104 | 135 | 0.22823 | -0.35427 | 1.472 |
| Eloc frontal | 13.413 | 11.842 | 1.1326 | 135 | 0.25937 | -10.007 | 36.833 |
| **PartCoef frontal*** | 24.518 | 10.102 | 2.4269 | 135 | 0.016547 | 4.5381 | 44.497 |
|  | | | | | | | |
| *Lack of perseverance ~ Limbic metrics* | | | | | | | |
|  | Estimate | SE | tStat | DF | pValue | Lower | Upper |
| (Intercept) | 24.842 | 5.7246 | 4.3395 | 136 | 2.77E-05 | 13.521 | 36.163 |
| ClusCoef limbic | -0.33943 | 4.9979 | -0.067915 | 136 | 0.94595 | -10.223 | 9.5442 |
| Deg limbic | 0.24734 | 0.21511 | 1.1498 | 136 | 0.25224 | -0.17806 | 0.67274 |
| Eccen limbic | 0.53339 | 0.66325 | 0.80421 | 136 | 0.42268 | -0.77822 | 1.845 |
| Eloc limbic | 6.2988 | 9.7344 | 0.64707 | 136 | 0.51868 | -12.951 | 25.549 |
| **PartCoef limbic*** | -26.553 | 11.396 | -2.33 | 136 | 0.021276 | -49.09 | -4.0169 |
|  | | | | | | | |
| *Sensation-seeking ~ Limbic metrics* | | | | | | | |
|  | Estimate | SE | tStat | DF | pValue | Lower | Upper |
| (Intercept) | 23.877 | 7.5021 | 3.1827 | 136 | 0.001808 | 9.0414 | 38.713 |
| ClusCoef limbic | 12.266 | 6.5497 | 1.8727 | 136 | 0.06326 | -0.68695 | 25.218 |
| Deg limbic | 0.30492 | 0.28191 | 1.0816 | 136 | 0.28133 | -0.25257 | 0.8624 |
| **Eccen limbic*** | 2.0243 | 0.86919 | 2.3289 | 136 | 0.021338 | 0.30538 | 3.7431 |
| Eloc limbic | -17.086 | 12.757 | -1.3394 | 136 | 0.18268 | -42.314 | 8.1413 |
| PartCoef limbic | 10.208 | 14.935 | 0.68349 | 136 | 0.49546 | -19.326 | 39.741 |

Table S5 - Significant Linear Mixed-effect Models (LMMs) predicting UPPS subscale scores from mean network metrics (i.e., ClusCoef: clustering coefficient; Deg: degree; Eccen: eccentricity; Eloc: local efficiency; PartCoef: participation coefficient) in younger individuals, divided according to frontal, limbic and striatal components (threshold: 40%)

### Older individuals 10%

| *Lack of premeditation ~ Limbic metrics* | | | | | | | |
| --- | --- | --- | --- | --- | --- | --- | --- |
|  | Estimate | SE | tStat | DF | pValue | Lower | Upper |
| (Intercept) | 19.561 | 1.2454 | 15.707 | 54 | 8.71E-22 | 17.065 | 22.058 |
| **ClusCoef limbic*** | 23.558 | 11.161 | 2.1107 | 54 | 0.039445 | 1.1811 | 45.936 |
| Deg limbic | 0.024648 | 0.89638 | 0.027497 | 54 | 0.97816 | -1.7725 | 1.8218 |
| Eccen limbic | 0.24 | 0.32179 | 0.74583 | 54 | 0.45901 | -0.40515 | 0.88515 |
| Eloc limbic | -21.502 | 11.784 | -1.8247 | 54 | 0.073587 | -45.127 | 2.1237 |
| PartCoef limbic | 4.9746 | 9.1785 | 0.54199 | 54 | 0.59006 | -13.427 | 23.376 |
|  | | | | | | | |
| *Lack of perseverance ~ Striatal metrics* | | | | | | | |
|  | Estimate | SE | tStat | DF | pValue | Lower | Upper |
| (Intercept) | 14.767 | 1.1603 | 12.727 | 54 | 6.92E-18 | 12.44 | 17.093 |
| **ClusCoef striatal*** | 10.71 | 5.2175 | 2.0527 | 54 | 0.044961 | 0.24949 | 21.17 |
| Deg striatal | 0.35199 | 0.60002 | 0.58664 | 54 | 0.55989 | -0.85097 | 1.555 |
| Eccent striatal | 0.34634 | 0.21943 | 1.5784 | 54 | 0.12031 | -0.09358 | 0.78627 |
| **Eloc striatal*** | -15 | 5.7333 | -2.6163 | 54 | 0.011503 | -26.495 | -3.5055 |
| PartCoef striatal | 7.8632 | 8.811 | 0.89243 | 54 | 0.37612 | -9.8018 | 25.528 |

Table S6 - Significant Linear Mixed-effect Models (LMMs) predicting UPPS subscale scores from mean network metrics (i.e., ClusCoef: clustering coefficient; Deg: degree; Eccen: eccentricity; Eloc: local efficiency; PartCoef: participation coefficient) in younger individuals, divided according to frontal, limbic and striatal components (threshold: 10%)

### Older individuals 30%

| *Lack of perseverance ~ Frontal metrics* | | | | | | | |
| --- | --- | --- | --- | --- | --- | --- | --- |
|  | Estimate | SE | tStat | DF | pValue | Lower | Upper |
| (Intercept) | 0.41285 | 9.548 | 0.04324 | 54 | 0.96567 | -18.73 | 19.555 |
| ClusCoef frontal | -4.1781 | 4.7563 | -0.87845 | 54 | 0.38359 | -13.714 | 5.3576 |
| **Deg frontal*** | -0.64481 | 0.29688 | -2.1719 | 54 | 0.034273 | -1.24 | -0.04959 |
| Eccen frontal | -0.33598 | 0.38696 | -0.86827 | 54 | 0.38909 | -1.1118 | 0.43982 |
| Eloc frontal | 20.27 | 12.008 | 1.6881 | 54 | 0.097155 | -3.8034 | 44.344 |
| PartCoef frontal | 23.82 | 13.904 | 1.7132 | 54 | 0.092406 | -4.055 | 51.695 |
|  | | | | | | | |
| *Sensation-seeking ~ Striatal metrics* | | | | | | | |
|  | Estimate | SE | tStat | DF | pValue | Lower | Upper |
| (Intercept) | 37.489 | 8.35 | 4.4897 | 55 | 3.69E-05 | 20.755 | 54.223 |
| ClusCoef striatal | -12.334 | 9.6885 | -1.2731 | 55 | 0.20834 | -31.75 | 7.0819 |
| Deg striatal | 0.41628 | 0.3788 | 1.0989 | 55 | 0.27659 | -0.34286 | 1.1754 |
| **Eccen striatal*** | -1.5565 | 0.7272 | -2.1404 | 55 | 0.036774 | -3.0138 | -0.09914 |
| Eloc striatal | 13.284 | 16.533 | 0.80353 | 55 | 0.42513 | -19.848 | 46.417 |
| PartCoef striatal | -18.407 | 21.064 | -0.87387 | 55 | 0.38599 | -60.621 | 23.806 |

Table S7 - Significant Linear Mixed-effect Models (LMMs) predicting UPPS subscale scores from mean network metrics (i.e., ClusCoef: clustering coefficient; Deg: degree; Eccen: eccentricity; Eloc: local efficiency; PartCoef: participation coefficient) in younger individuals, divided according to frontal, limbic and striatal components (threshold: 30%)

### Older individuals 40%

| *Urgency ~ Frontal metrics* | | | | | | | |
| --- | --- | --- | --- | --- | --- | --- | --- |
|  | Estimate | SE | tStat | DF | pValue | Lower | Upper |
| (Intercept) | 6.9706 | 15.317 | 0.45508 | 51 | 0.65099 | -23.78 | 37.721 |
| **ClusCoef frontal*** | -12.048 | 5.1149 | -2.3556 | 51 | 0.022377 | -22.317 | -1.78 |
| Deg frontal | -0.56978 | 0.31502 | -1.8087 | 51 | 0.076392 | -1.2022 | 0.062647 |
| Eccen frontal | -0.78697 | 0.56804 | -1.3854 | 51 | 0.17195 | -1.9274 | 0.35341 |
| **Eloc frontal*** | 39.447 | 18.383 | 2.1458 | 51 | 0.036667 | 2.5416 | 76.353 |
| PartCoef frontal | 6.8357 | 17.443 | 0.39189 | 51 | 0.69677 | -28.183 | 41.854 |
|  | | | | | | | |
| *Sensation-seeking ~ Frontal metrics* | | | | | | | |
|  | Estimate | SE | tStat | DF | pValue | Lower | Upper |
| (Intercept) | -10.442 | 24.543 | -0.42546 | 54 | 0.67219 | -59.647 | 38.763 |
| ClusCoef frontal | 14.218 | 8.2195 | 1.7298 | 54 | 0.089372 | -2.2607 | 30.698 |
| Deg frontal | 0.30946 | 0.49911 | 0.62002 | 54 | 0.53785 | -0.6912 | 1.3101 |
| **Eccen frontal*** | 2.0652 | 0.88475 | 2.3342 | 54 | 0.023336 | 0.29136 | 3.839 |
| Eloc frontal | -1.7097 | 29.421 | -0.05811 | 54 | 0.95387 | -60.694 | 57.275 |
| PartCoef frontal | 29.914 | 27.763 | 1.0775 | 54 | 0.28605 | -25.747 | 85.575 |

Table S8 - Significant Linear Mixed-effect Models (LMMs) predicting UPPS subscale scores from mean network metrics (i.e., ClusCoef: clustering coefficient; Deg: degree; Eccen: eccentricity; Eloc: local efficiency; PartCoef: participation coefficient) in younger individuals, divided according to frontal, limbic and striatal components (threshold: 40%)

## Testing robustness of main results with different parcellation scheme

To test the robustness of our results, we re-ran our analyses using a different parcellation scheme based on the Yeo 7-Network atlas (Yeo et al., 2011), consisting of 51 ROIs. A total of 18 ROIs (14 frontal, 4 limbic) were used to replicate our results on the association between graph-theoretical measures and impulsivity scores.

As shown in Table S9-S10, results from the linear mixed-effect models show a similar pattern of increased integration and efficiency, as well as of decreased segregation for younger impulsive individuals. Furthermore, this pattern was reversed for older impulsive individuals (Table S10), in line with what reported in the main manuscript with the Schaefer atlas.

| *Lack of premeditation ~ Frontal metrics* | | | | | | | |
| --- | --- | --- | --- | --- | --- | --- | --- |
|  | Estimate | SE | tStat | DF | pValue | Lower | Upper |
| (Intercept) | 26.161 | 1.9946 | 13.116 | 134 | 8.52E-26 | 22.216 | 30.106 |
| **ClusCoef frontal*** | -9.2153 | 4.2668 | -2.1598 | 134 | 0.032569 | -17.654 | -0.77637 |
| Deg frontal | -0.67588 | 0.56301 | -1.2005 | 134 | 0.23207 | -1.7894 | 0.43765 |
| Eccen frontal | -0.33612 | 0.35619 | -0.94366 | 134 | 0.34704 | -1.0406 | 0.36836 |
| **Eloc frontal*** | 11.067 | 4.8445 | 2.2844 | 134 | 0.023922 | 1.485 | 20.648 |
| PartCoef frontal | -7.2682 | 6.24 | -1.1648 | 134 | 0.24618 | -19.61 | 5.0734 |
|  | | | | | | | |
| *Lack of premeditation ~ Limbic metrics* | | | | | | | |
|  | Estimate | SE | tStat | DF | pValue | Lower | Upper |
| (Intercept) | 23.936 | 1.0765 | 22.235 | 134 | 8.39E-47 | 21.807 | 26.065 |
| **ClusCoef limbic*** | -9.4831 | 3.3505 | -2.8304 | 134 | 0.005366 | -16.11 | -2.8564 |
| Deg limbic | -0.53905 | 0.34158 | -1.5781 | 134 | 0.1169 | -1.2146 | 0.13655 |
| Eccen limbic | -0.087893 | 0.23631 | -0.37193 | 134 | 0.71053 | -0.55528 | 0.37949 |
| **Eloc limbic*** | 9.2824 | 3.6293 | 2.5576 | 134 | 0.011653 | 2.1042 | 16.461 |
| PartCoef limbic | 6.9611 | 5.1949 | 1.34 | 134 | 0.18251 | -3.3134 | 17.236 |
|  | | | | | | | |
| *Lack of perseverance ~ Limbic metrics* | | | | | | | |
|  | Estimate | SE | tStat | DF | pValue | Lower | Upper |
| (Intercept) | 16.969 | 1.3429 | 12.636 | 135 | 1.21E-24 | 14.314 | 19.625 |
| ClusCoef limbic | 7.6093 | 4.1606 | 1.8289 | 135 | 0.069619 | -0.61899 | 15.838 |
| Deg limbic | 0.20839 | 0.43179 | 0.48262 | 135 | 0.63015 | -0.64556 | 1.0623 |
| **Eccen limbic*** | 0.60787 | 0.29606 | 2.0532 | 135 | 0.041981 | 0.022364 | 1.1934 |
| Eloc limbic | -7.4808 | 4.5046 | -1.6607 | 135 | 0.099095 | -16.39 | 1.4279 |
| PartCoef limbic | 7.2228 | 6.5387 | 1.1046 | 135 | 0.27129 | -5.7088 | 20.154 |
|  | | | | | | | |
| *Sensation-seeking ~ Limbic metrics* | | | | | | | |
|  | Estimate | SE | tStat | DF | pValue | Lower | Upper |
| (Intercept) | 32.525 | 1.7915 | 18.155 | 136 | 3.66E-38 | 28.982 | 36.068 |
| ClusCoef limbic | 4.8605 | 5.5399 | 0.87737 | 136 | 0.38183 | -6.095 | 15.816 |
| Deg limbic | 0.3008 | 0.57044 | 0.52731 | 136 | 0.59884 | -0.82728 | 1.4289 |
| **Eccen limbic*** | 0.95595 | 0.39482 | 2.4212 | 136 | 0.016787 | 0.17517 | 1.7367 |
| Eloc limbic | -9.5714 | 6.0065 | -1.5935 | 136 | 0.11337 | -21.45 | 2.3068 |
| PartCoef limbic | 8.9204 | 8.6737 | 1.0284 | 136 | 0.30557 | -8.2324 | 26.073 |

Table S9 - Significant Linear Mixed-effect Models (LMMs) predicting UPPS subscale scores from mean network metrics (i.e., ClusCoef: clustering coefficient; Deg: degree; Eccen: eccentricity; Eloc: local efficiency; PartCoef: participation coefficient) in younger individuals, divided according to frontal, limbic and striatal components (threshold: 80%) as derived from the Yeo 7-Network cortical atlas (Yeo et al., 2011)

| *Sensation-seeking ~ Frontal metrics* | | | | | | | |
| --- | --- | --- | --- | --- | --- | --- | --- |
|  | Estimate | SE | tStat | DF | pValue | Lower | Upper |
| (Intercept) | 32.902 | 4.5867 | 7.1732 | 54 | 2.16E-09 | 23.706 | 42.098 |
| ClusCoef frontal | -7.8587 | 10.754 | -0.73079 | 54 | 0.46807 | -29.419 | 13.701 |
| Deg frontal | 1.3618 | 1.1908 | 1.1436 | 54 | 0.25783 | -1.0256 | 3.7492 |
| **Eccen frontal*** | -1.6086 | 0.70509 | -2.2814 | 54 | 0.026493 | -3.0222 | -0.19498 |
| Eloc frontal | 4.0453 | 11.712 | 0.34541 | 54 | 0.73113 | -19.435 | 27.526 |
| PartCoef frontal | -17.629 | 12.807 | -1.3766 | 54 | 0.17433 | -43.305 | 8.0467 |
|  | | | | | | | |
| *Lack of premeditation ~ Limbic metrics* | | | | | | | |
|  | Estimate | SE | tStat | DF | pValue | Lower | Upper |
| (Intercept) | 22.033 | 1.1232 | 19.615 | 54 | 3.13E-26 | 19.781 | 24.285 |
| ClusCoef limbic | 5.6128 | 4.7334 | 1.1858 | 54 | 0.2409 | -3.877 | 15.103 |
| Deg limbic | -0.74323 | 0.56108 | -1.3246 | 54 | 0.19087 | -1.8681 | 0.38167 |
| Eccen limbic | -0.41823 | 0.23578 | -1.7738 | 54 | 0.081736 | -0.89095 | 0.054486 |
| Eloc limbic | -0.61364 | 5.1322 | -0.11957 | 54 | 0.90527 | -10.903 | 9.6758 |
| **PartCoef limbic*** | 13.748 | 4.7449 | 2.8974 | 54 | 0.0054245 | 4.2351 | 23.261 |

Table S10 - Significant Linear Mixed-effect Models (LMMs) predicting UPPS subscale scores from mean network metrics (i.e., ClusCoef: clustering coefficient; Deg: degree; Eccen: eccentricity; Eloc: local efficiency; PartCoef: participation coefficient) in older individuals, divided according to frontal, limbic and striatal components (threshold: 80%) as derived from the Yeo 7-Network cortical atlas (Yeo et al., 2011)
